# Air movement sound production by alewife, white sucker, and four salmonid fishes suggests the phenomenon is widespread among freshwater fishes

**DOI:** 10.1371/journal.pone.0204247

**Published:** 2018-09-20

**Authors:** Rodney A. Rountree, Francis Juanes, Marta Bolgan

**Affiliations:** 1 The Fish Listener, East Falmouth, Massachusetts, United States of America; 2 Biology Department, University of Victoria, Victoria, BC, Canada; 3 Laboratoire de Morphologie Fonctionnelle et Evolutive, Université de Liège, Liège, Belgium; University of South Florida, UNITED STATES

## Abstract

We sought to describe sounds of some of the common fishes suspected of producing unidentified air movement sounds in soundscape surveys of freshwater habitats in the New England region of North America. Soniferous behavior of target fishes was monitored in real time in the field in both natural and semi-natural environments by coupling Passive Acoustic Monitoring (PAM) with direct visual observation from shore and underwater video recording. Sounds produced by five species including, alewife (*Alosa pseudoharengus*, Clupeidae), white sucker (*Catastomus commersonii*, Catostomidae), brook trout (*Salvelinus fontinalis*, Salmonidae), brown trout (*Salmo trutta*, Salmonidae), and rainbow trout (*Oncorhynchus mykiss*, Salmonidae) were validated and described in detail for the first time. In addition, field recordings of sounds produced by an unidentified salmonid were provisionally attributed to Atlantic salmon (*Salmo salar*, Salmonidae). Sounds produced by all species are of the air movement type and appear to be species specific. Our data based on fishes in three distinct orders suggest the phenomenon may be more ecologically important than previously thought. Even if entirely incidental, air movement sounds appear to be uniquely identifiable to species and, hence, hold promise for PAM applications in freshwater and marine habitats.

## Introduction

Passive acoustic monitoring (PAM) has become an important tool for spatial and temporal location of marine fishes based on their incidental and/or purposeful sound production [1–2]. However, little is known about sound production by freshwater fishes in the New England region of North America [3]. While conducting a pilot survey of the soundscapes of freshwater habitats in five regions of New England in the spring of 2008, we recorded possible air movement sounds likely produced by alewife (*Alosa pseudoharengus*, Clupeidae) and various species of unidentified salmonids [4]. We also recorded a wide variety of Fast Repetitive Tick (FRT) sounds [5] from unknown sources with different tick decay patterns (rate of increase in the interval between ticks), durations and frequency ranges. However, we could not validate the identity of the sound sources based on the data collected at that time. We use the term “air movement” sound to include a diverse array of sounds sometimes referred to as “air passage” or “pneumatic” sounds that arise from a variety of mechanisms involving internal air movement, and sometimes external air release, in physostomous fishes (e.g., [6–9]).

The fundamental goal of this paper was to confirm that the widespread occurrence of air movement-like sounds previously recorded in the 2008 field survey [4] were indeed produced by fishes, and to describe specific sounds produced by species suspected of producing them. The specific aims of this study were to: i) describe the acoustic characteristics and the behavioral association of air movement sounds in the alewife, the white sucker (*Catastomus commersonii*, Catostomidae), and the brook, brown and rainbow trout (*Salvelinus fontinalis*, *Salmo trutta*, and *Oncorhynchus mykiss*, Salmonidae, respectively) and ii) compare the acoustic features of air movement sounds emitted by these species to determine if they are species specific, and thus potentially useful in ecological studies based on PAM.

## Materials and methods

### Sampling locations and species identification

Fish behavior was observed in real time in the field in natural and semi-natural environments by coupling PAM with direct visual observation from shore and with underwater video recording. The species-specific identity of the sound emitter was validated through direct observation or because observations were carried out where only one species was present. Fishes were observed in private locations with permission from the owners and public locations that did not require permissions. Animals were observed remotely and not disturbed in any way. No permitting or ethics oversight approval was necessary. Field observations were made on wild fish in seven locations within Massachusetts and Maine, and on captive fish held in semi-natural conditions at the Blue Stream Aquaculture facility in Barnstable, Massachusetts (detailed description of sampling locations and methods are provided in S1 Appendix). Sounds were recorded with either an uncalibrated HTI-96-MIN (High Tech Industries, Gulfport, MS; sensitivity = -165 dB re: 1 V/μPa, frequency response: 2 Hz to 30 kHz) or SQ26-08 (Sensitivity = -169.00 re. 1V/μPa rms, Cetacean Research Technology, Seattle, WA) hydrophone. Ambient aerial sounds and voice notes were recorded to a second channel from a microphone to verify if unknown sounds originated underwater. Underwater video was observed with one or more underwater cameras mounted in a fixed location on the bottom or suspended within the water column. Post-processing of acoustic signals was conducted by listening to all recordings in their entirety while simultaneously viewing the sound’s spectrogram (1,024 FFT, Hanning window, 50% overlap) and waveform with Raven Pro 1.5 acoustic software [10].

Sounds were categorized into three types: 1) surface event, 2) fish sounds, and 3) air bubble sounds. Surface events were identified when a fish rose to the surface to gulp air often creating a splash or jump sound. Although such sounds are not generally considered as “fish sounds” we consider them a critical component of air movement sound production behavior and potentially useful in fish identification and informative of behavior and physiological differences among species. Sounds produced by the fish following the surface event were classified as fish sounds, except for air bubble sounds which were difficult to detect and appeared to be incidental to sound production. Acoustic measurements of selected parameters of all sound types were made in Raven following Charif et al. [11]. Parameter definitions are provided in S1 Appendix.

**Fig 1:**
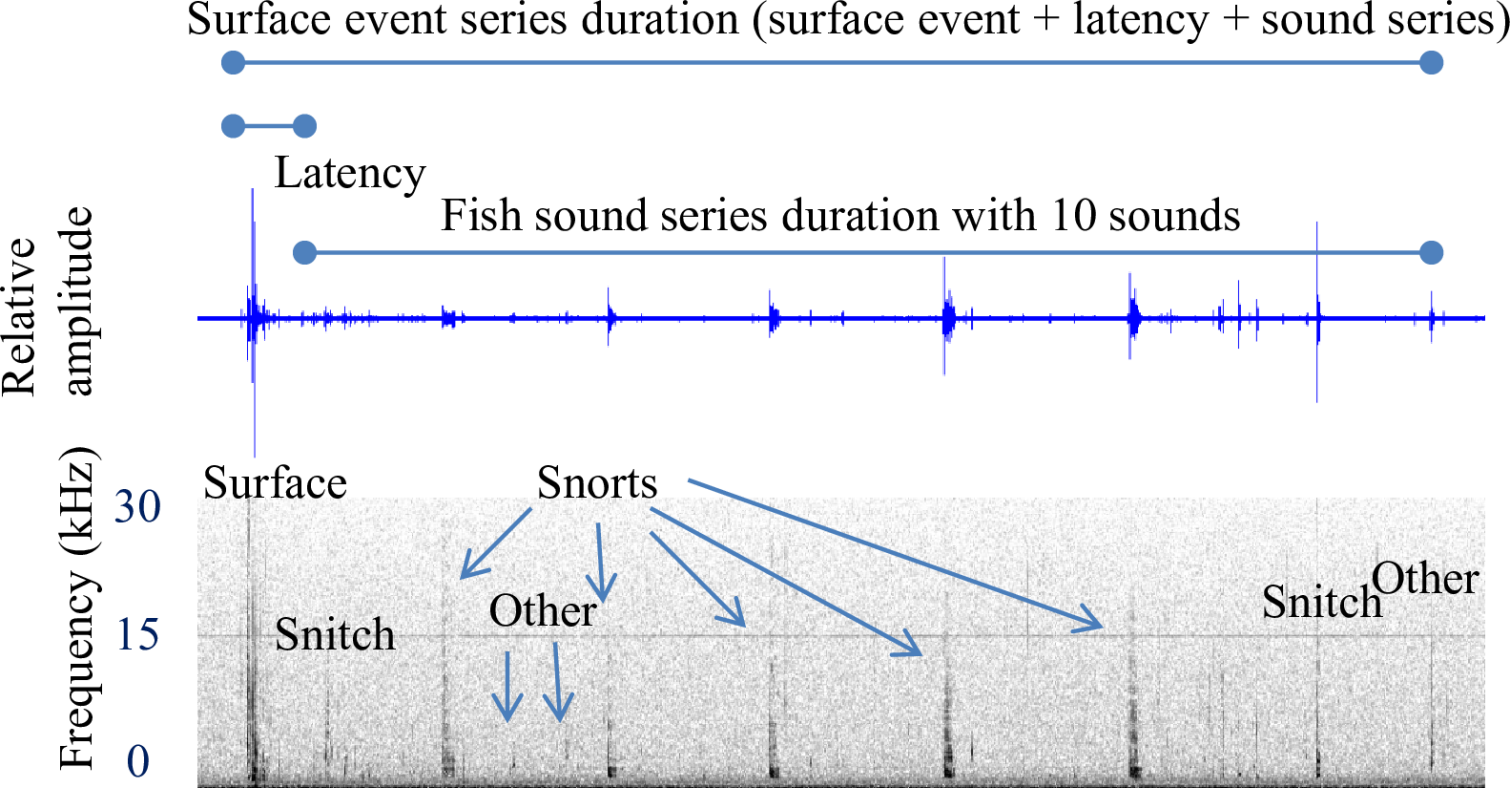
Sound series measurement definitions. The surface event sound series includes the surface event, latency, fish sound series, and incidental bubble sounds. In this example from a white sucker, the fish sound series consists of 11 sounds (2 snitches, 3 other, and 5 snorts). The surface event series duration is measured from the start of the surface event to the end of the last fish sound. Latency is measured as the duration from the start of the surface event to the start of the first fish sound. In this example, the latency is measured from the start of the surface event to the start of the “snitch” sound. The fish sound series duration is measured between the start of the first fish sound to the end of the last fish sound. Note, that when the surface event could not be detected, only the fish sound series measurements could be made.

It was sometimes possible to attribute a continuous train of sounds as a “fish sound series” produced by a single fish, either through direct observation, or temporal isolation from other sounds. The fish sound series is analogous to a fish call series, but we refrain from labeling it as such because that implies a communication function which has not been established. In some cases, we were able to identify the surface event preceding the fish sound series and recognized a “surface event sound series” which includes the fish sound series and is also attributable to a single fish. For each fish sound series, the following metrics were measured (Fig 1): i) number of individual sounds (excluding bubble sounds); ii) duration (the time between the start of the first sound to the end of the last sound; iii) sound rate (the number of sounds in a fish sound series divided by the series duration), and iv) sound period (series duration divided by the number of sounds). For the surface event sound series, the following additional metrics were measured; i) duration (the time between the start of the surface event to the end of the last fish sound); ii) latency (the time elapsed between the start of the surface event and the start of the first fish sound).

To provide additional insight into the sound production mechanisms, the pulse structure of known bubble sounds was compared with that of individual ticks of FRT sounds recorded during this study, and with those of Pacific herring (*Clupea pallasii*, Clupeidae) recorded with the same type of hydrophone and recording system (sound samples provided by Amalis Riera, University of Victoria, Canada).

### Statistical analysis

To provide the best descriptions of individual sound types, data were pooled over all measurements for each species because many more sounds were observed than could be reliably attributed to a single sound series. However, the determination of the most common sounds observed for each species was based only on the subset of sounds that could be attributed to individual fish sound series, thus it represents the types of sounds most likely to be produced by the species rather than the most numerous sound type.

In order to explore the potential of PAM for surveying specific species based on air movement sounds, selected acoustic parameters were statistically compared among species. We tested for univariate among-species differences in sound series parameters (surface event series duration, latency, fish sound series duration, number of fish sounds, fish sound period and rate) with a one-way analysis of variance (ANOVA) for each variable using SAS/STAT software, Version 12.1 [12]. Similarly, we tested for among-species differences in selected sound parameters for the most common sound types. Different sets of species were included in the analyses depending on the sound type, as many sounds were not shared among species. For both sets of analyses variables were first normalized with the most appropriate transformation based on a maximum likelihood test. In a few cases statistical outliers were omitted.

Canonical Discrimination Analysis (CDA) and Multivariate Analysis of Variance (MANOVA) were used to test for multivariate differences in acoustic parameters among species for the most common sound types after first conducting a stepwise discriminant analysis to select a subset of the variables for group discrimination [12–15]. The CDA tests whether species can be distinguished by a sound type, and if so, which acoustic parameters contribute the most to the observed differences (for example, a CDA on the surface event sound compares the acoustic parameters of the surface event sound among all species, since all exhibited that type of sound, however, the CDA on bubble sounds only compares the three species that exhibited them). A second CDA analysis compared all species by including all sounds they exhibited, and thus takes into account differences in sound types among species. Pearson correlations of the original (but transformed to normalize) variables with the derived canonical variables from the CDA analyses were calculated to determine which sound parameter contributed most to the group discrimination [14–15].

## Results

A total of 56.5 h (3,388 min) of observations were made, in which we measured the acoustic parameters for 1,195 sounds attributed to the study species (S1 Appendix). A total of 160 fish sound series were identified of which 117 had associated surface events (so there were 117 surface event series, Table 1).

**Table 1:** Sound series statistics.

Species	Statistic	Surface event series duration	Latency	Fish sound series duration	Number of fish sounds	Fish sound rate	Fish sound period
Alewife	N	21	21	33	33	33	33
	min	1.15	0.35	0.08	1.00	0.50	0.08
	max	11.66	4.14	8.45	16.00	12.05	2.01
	SE	0.54	0.21	0.33	0.61	0.38	0.06
	mean	5.39	1.19	3.29	5.94	2.47	0.57
White sucker	N	11	11	12	12	12	12
	min	8.02	1.47	1.73	3.00	0.28	0.58
	max	28.62	4.46	24.89	11.00	1.73	3.56
	SE	1.90	0.24	2.07	0.66	0.12	0.26
	mean	20.52	2.58	16.75	7.08	0.56	2.33
Brook trout	N	4	4	6	6	6	6
	min	0.83	0.45	0.14	1.00	0.66	0.14
	max	12.35	12.08	4.57	3.00	7.35	1.52
	SE	2.88	2.98	0.70	0.33	0.90	0.21
	mean	6.62	6.24	1.12	1.67	3.36	0.50
Brown trout	N	4	4	26	26	26	26
	min	0.36	0.29	0.07	1.00	0.57	0.07
	max	9.36	8.38	5.23	3.00	13.33	1.74
	SE	1.88	1.78	0.23	0.11	0.71	0.09
	mean	4.14	3.31	1.31	2.19	3.85	0.56
Rainbow trout	N	42	42	42	42	42	42
	min	0.75	0.32	0.06	1.00	0.26	0.06
	max	7.20	6.50	3.81	7.00	16.67	3.81
	SE	0.23	0.20	0.15	0.20	0.55	0.09
	mean	2.77	1.68	1.09	1.93	3.87	0.56
Unknown salmonid	N	35	35	41	41	41	41
	min	0.81	0.45	0.11	1.00	0.48	0.11
	max	19.25	4.66	17.30	10.00	9.43	2.10
	SE	0.51	0.19	0.45	0.30	0.23	0.08
	mean	4.22	1.63	2.88	2.93	1.56	0.95
MANOVA		***	**	***	***	***	***
Means comparison		WS > all	WS > all	WS > all	WS = A > all	WS > all	WS > all
Sample size		117	117	160	160	160	160

Comparison of sound series parameters among species. MANOVA = results of a Multivariate Analysis of Variance

* = p < 0.05

** = p < 0.01

*** = p < 0.0001.

WS = white sucker, A = alewife, all = remainder species. N = sample size, min = minimum, max = maximum, SE = standard error of the mean

### Alewife

Over 30 h of observations (1,857 min) were made of alewife behavior (S1 Appendix), however to reduce the possibility of inclusion of sounds from blueback herring (*Alosa aestivalis*, Clupeidae), we excluded sound data collected after May 2 from the measurement data. Alewife observations were usually done during the afternoon daylight hours through the early evening. Typically, from a few to a dozen or more individuals occupied the observation chambers at any given time in both the Mill Creek and Webber Pond raceways as they rested prior to moving into the adjacent ponds. The mill pool typically held from dozens to hundreds of alewives. In contrast, Sevenmile Brook below the Webber Pond dam held thousands of fish. Fish appeared to segregate into small groups, or “pods”, of up to a dozen individuals.

**Fig 2:**
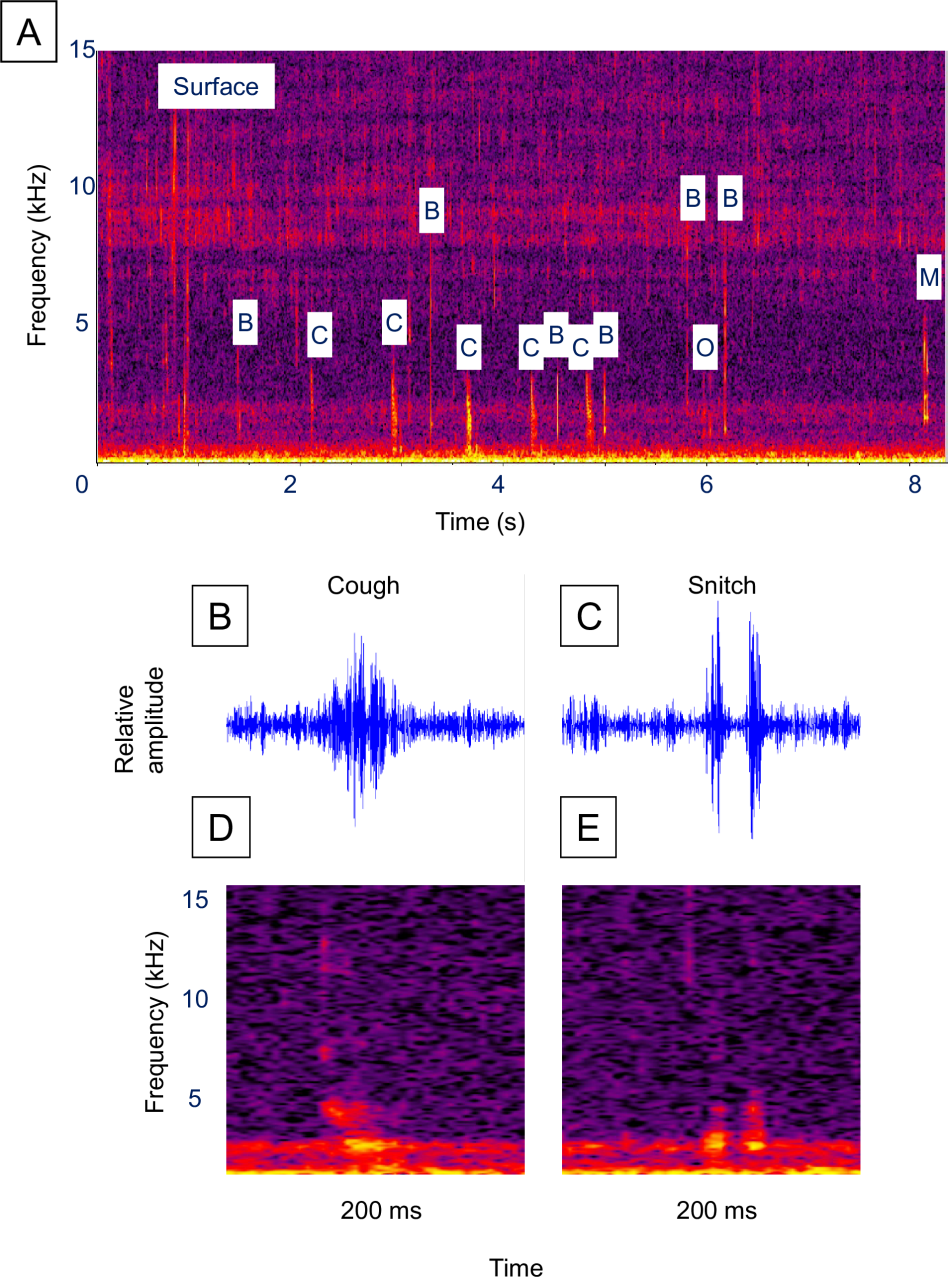
Alewife sounds. Example of a surface event sound series and the two most frequently observed fish sounds. (**A)** Spectrogram of a surface event sound series (S1 Audio, S1 and S2 Videos), (**B)** waveform of a cough, (**C)** waveform of a snitch, (**D)** spectrogram of the cough in **B**, and (**E)** spectrogram of the snitch in **C**. Figure labels: “Surface” = air gulping surface event, “B” = bubble sound, “C” = cough fish sound, “O” = other fish sound, “M” = mouth bubble sound. Spectrogram parameters: unfiltered, 1,024-point Hann windowed FFTs with 50% overlap. Fish sound waveforms were filtered around 600 Hz to 4000 Hz.

Alewife were frequently observed to make a series of sounds after rising to the surface to gulp air (Fig 2A, S1 Audio, S1 and S2 Videos). Individuals would make a rapid dash to the surface and create a small splash, occasionally jumping, as they gulped air. They then rapidly dove to resume swimming at the same depth they started from. Sounds were primarily produced after resuming their previous swimming depth. Of 54 surfacing events recorded in field notes, 34 (63%) were followed by sound production. In many cases silent bubbles escaped from the mouth and gills during the initial descent from the surface. However, once the fish sounds began, a faint “pop” sound could sometimes be detected as one or more bubbles escaped from the gills after each sound. On a few occasions, a sound series concluded with a loud pop as a single large bubble was released from the mouth (Fig 2A, S1 Video). The bubble sounds were difficult to detect and were distinct from high frequency burst sounds produced by the alewife which were labeled “coughs” and “snitches”. Although the cough and snitch sounds were associated with air bubble release, air bubble release alone did not produce them. For example, four of 19 field observations of air bubble release resulted in no detectable sound production (21%). We therefore considered these faint bubble sounds to be incidental to the production of the cough and snitch sounds and excluded them from the fish sound series. Although readily visible to an observer from shore, these events were very difficult to capture on video. However, we were successful in capturing video of alewife sound production (S1 Audio, S1 and S2 Videos), as well as air bubble release without detectable sound production (S3 and S4 Videos) on several occasions. No obvious reaction of conspecifics to alewife sound production was observed.

Alewife sound series had an average of 5.9 fish sounds (range 1–16) and a mean fish sound rate of 2.47 sounds/s (Table 1). Alewife surface event series duration averaged 5.39 s while the latency before sound production ranged from 0.35 to 4.14 s and averaged 1.19 s. The most frequently occurring sounds in an alewife sound series (Table 2) were the “cough” (91%) and “snitch” (21%). Air bubbles were not included as part of the sound series but were acoustically detected in 20 series (61%) and occurred an average of 0.36 s after each cough or snitch (range 0.026–0.986 s, standard error = 0.034 s). Coughs had a mean peak frequency of 1,258 Hz, bandwidth of 3,609 Hz and duration of 0.043 s (Fig 2A, 2B and 2D, Table 3).

**Table 2:** Most common sounds.

Sound type	Frequency	Percent
**Alewife N = 33**		
Cough	30	91
Snitch	7	21
Other	5	15
FRT-like	3	9
Snort	2	6
Surface	21	64
Bubble	20	61
Mouth bubble	2	6
**White sucker N = 12**		
Snort	12	100
Other	4	33
Snitch	4	33
FRT-like	1	8
Sneeze	1	8
Surface	11	92
Bubble	7	58
**Brook trout N = 6**		
VFRT	3	50
Other	3	50
Snitch	2	33
FRT long	1	17
Snort	1	17
Surface	4	67
**Brown trout N = 26**		
VFRT	25	96
Other	8	31
Chirp	7	27
Bubble FRT	1	4
200 Hz FRT	1	4
Splash	4	15
Bubble	1	4
**Rainbow trout N = 42**		
Gurgle	23	55
Other	9	21
VFRT	7	17
Snitch	7	17
Chirp	3	7
Long FRT	3	7
FRT-like	2	5
Surface	42	100
**Unknown salmonid N = 41**		
Moan	14	34
Other	13	32
Snort FRT	6	15
FRT-like bubbles	5	12
FRT-like	4	10
Snort	4	10
Jump	35	85
Bubbles	10	24

Sound types most frequently exhibited by a species, including surface event and bubble types, based on measurements from fish sound series (N = number of sound series, FRT = fast repetitive tick, VFRT = very fast repetitive tick)

**Table 3:** Acoustic characteristics of the common sounds.

		Frequency statistics									
Type	Sample size	Low	1st quartile	Peak	Center	3rd quartile	High	90% bandwidth	bandwidth	90% duration	Duration
**Alewife**											
Cough	154	603 (36)	1064 (48)	1258 (61)	1489 (60)	1979 (77)	4212 (149)	2183 (106)	3609 (140)	0.03 (0.002)	0.043 (0.003)
Snitch	27	733 (98)	1243 (132)	1434 (172)	1529 (166)	2069 (249)	3682 (396)	1755 (297)	2949 (358)	0.014 (0.004)	0.02 (0.005)
Other	9	817 (106)	1796 (191)	1864 (183)	2197 (228)	2750 (364)	4966 (744)	2614 (581)	4149 (708)	0.062 (0.024)	0.094 (0.036)
FRT-like	7	2884 (704)	4258 (1192)	6254 (2121)	5872 (1830)	7700 (2563)	11641 (4305)	7031 (3011)	8756 (3660)	1.234 (0.558)	1.373 (0.610)
Snort	7	463 (115)	1118 (212)	1198 (272)	1392 (255)	2156 (473)	5320 (709)	2571 (631)	4857 (690)	0.066 (0.005)	0.096 (0.011)
Unknown	5	1207 (246)	2418 (349)	3253 (901)	3290 (880)	4068 (1120)	9497 (3892)	5287 (2559)	8289 (3844)	0.618 (0.437)	0.745 (0.502)
Splash	19	747 (164)	1741 (374)	1939 (261)	2407 (471)	3658 (662)	10189 (2427)	4894 (794)	9441 (2422)	0.204 (0.050)	0.262 (0.055)
Bubble	59	890 (58)	1571 (99)	1716 (140)	1966 (145)	2412 (169)	4410 (285)	1917 (216)	3520 (272)	0.003 (0.001)	0.009 (0.001)
Mouth bubble	2	1596 (281)	1875 (187)	1992 (304)	2320 (117)	3140 (796)	4253 (1563)	1664 (1054)	2656 (1844)	0.139 (0.107)	0.169 (0.133)
**White sucker**											
Snort	60	756 (63)	1534 (111)	1954 (134)	2260 (165)	3554 (266)	13042 (1414)	5829 (613)	12285 (1428)	0.13 (0.012)	0.175 (0.017)
Snitch	9	929 (219)	1791 (225)	2291 (279)	2385 (264)	3875 (552)	11277 (1308)	5145 (572)	10348 (1338)	0.06 (0.020)	0.083 (0.021)
Sneeze	5	840 (121)	3956 (1801)	5868 (3002)	6562 (2992)	12037 (4422)	46746 (185)	26943 (2665)	45906 (118)	0.116 (0.021)	0.131 (0.025)
Other	3	941 (267)	1500 (162)	2218 (125)	2062 (143)	2468 (136)	3250 (198)	1656 (250)	2308 (176)	0.046 (0.005)	0.059 (0.008)
Splash	6	312 (185)	718 (289)	1187 (292)	1218 (322)	2343 (1003)	26436 (7220)	5828 (2199)	26123 (7381)	0.248 (0.090)	0.391 (0.122)
Jump	5	442 (192)	1237 (439)	1949 (826)	1725 (645)	2437 (777)	24730 (9148)	5100 (1215)	24288 (9299)	0.271 (0.133)	0.438 (0.159)
Bubble	23	808 (133)	1406 (208)	1548 (221)	1581 (205)	2013 (191)	3603 (226)	1288 (243)	2794 (225)	0.007 (0.002)	0.014 (0.002)
Bubble like	15	279 (25)	450 (33)	687 (185)	750 (165)	1156 (193)	2454 (358)	1518 (303)	2174 (370)	0.031 (0.008)	0.038 (0.008)
**Brook trout**											
VFRT	19	2378 (141)	4332 (377)	4993 (400)	5531 (696)	6962 (1072)	12785 (2046)	6809 (1417)	10407 (2024)	0.058 (0.005)	0.096 (0.008)
Snitch	12	2654 (288)	4023 (382)	4617 (588)	4773 (498)	5664 (593)	7820 (878)	3742 (610)	5166 (770)	0.074 (0.010)	0.099 (0.012)
Chirp	4	3673 (1307)	4382 (1406)	5296 (1560)	4992 (1424)	5648 (1313)	6485 (1422)	2109 (728)	2811 (735)	0.131 (0.040)	0.2 (0.051)
FRT	3	2295 (663)	4312 (614)	3562 (248)	5593 (1104)	6656 (1647)	11997 (3063)	6406 (2890)	9702 (3228)	0.297 (0.117)	0.378 (0.083)
Snort	3	1888 (324)	2843 (706)	2937 (773)	3031 (706)	3406 (596)	4383 (894)	1843 (450)	2495 (570)	0.112 (0.036)	0.177 (0.051)
Splash	7	2214 (668)	4888 (771)	5758 (857)	6977 (1041)	9321 (1786)	26683 (6024)	15696 (3312)	24469 (6159)	0.181 (0.034)	0.239 (0.045)
**Brown trout**											
VFRT	216	2568 (60)	4187 (84)	4582 (101)	5056 (104)	6131 (137)	12260 (471)	5191 (218)	9692 (464)	0.078 (0.003)	0.111 (0.004)
Chirp	9	2279 (210)	3729 (199)	4760 (436)	5000 (248)	6697 (425)	9333 (714)	5479 (562)	7054 (748)	0.065 (0.010)	0.082 (0.012)
Other	7	2834 (756)	4151 (979)	4566 (1043)	4781 (970)	5625 (1005)	8138 (1302)	3455 (724)	5303 (940)	0.12 (0.027)	0.146 (0.026)
Unknown	7	1195 (446)	1794 (480)	2035 (533)	2544 (794)	3656 (1448)	5222 (1757)	2892 (1178)	4027 (1431)	0.846 (0.527)	0.979 (0.598)
Splash	11	1340 (315)	4926 (906)	7678 (3046)	7747 (1920)	11786 (3144)	42311 (2794)	19321 (3481)	40970 (2769)	0.241 (0.071)	0.373 (0.086)
Bubble	3	431 (215)	843 (324)	1031 (378)	1281 (360)	1562 (409)	9861 (1801)	1750 (307)	9430 (1585)	0.004 (0.002)	0.016 (0.002)
**Rainbow trout**											
Gurgle	54	1442 (51)	2175 (57)	2409 (86)	2505 (74)	2954 (98)	3911 (146)	1779 (107)	2468 (142)	0.278 (0.039)	0.337 (0.042)
VFRT	19	2089 (209)	3651 (378)	4199 (533)	4741 (667)	6024 (949)	12775 (2745)	6029 (1347)	10686 (2722)	0.088 (0.016)	0.129 (0.025)
Snitch	14	1969 (133)	3187 (332)	3877 (440)	3850 (451)	5176 (733)	8903 (1700)	4794 (1068)	6934 (1632)	0.05 (0.009)	0.068 (0.010)
Chirp	10	1875 (94)	2531 (99)	2793 (180)	2953 (168)	3674 (388)	5320 (711)	2737 (638)	3444 (746)	0.195 (0.069)	0.247 (0.081)
Other	8	1309 (147)	2027 (101)	2062 (217)	2601 (214)	3363 (482)	4855 (988)	2660 (894)	3546 (1002)	0.137 (0.030)	0.185 (0.035)
Splash	49	1573 (55)	3587 (170)	4302 (338)	5491 (313)	8274 (364)	37069 (1621)	15059 (660)	35495 (1638)	0.268 (0.018)	0.399 (0.024)
**Unknown salmonid**											
Gurgle	58	421 (19)	662 (24)	748 (29)	796 (28)	934 (35)	1694 (62)	745 (41)	1272 (67)	0.313 (0.043)	0.401 (0.051)
Snort	32	495 (12)	729 (10)	829 (26)	820 (13)	949 (25)	1691 (66)	638 (47)	1195 (66)	0.111 (0.011)	0.141 (0.013)
Moan	15	445 (35)	818 (88)	962 (122)	943 (110)	1087 (110)	1983 (319)	756 (127)	1538 (310)	0.338 (0.052)	0.438 (0.066)
Other	13	467 (69)	923 (114)	1031 (129)	1247 (146)	1600 (160)	7273 (3429)	1838 (319)	6805 (3467)	0.326 (0.162)	0.571 (0.318)
Snort FRT	10	375 (65)	1031 (147)	1218 (203)	1218 (155)	1640 (213)	4111 (520)	1593 (226)	3736 (547)	0.142 (0.025)	0.19 (0.029)
Snitch	6	1004 (204)	1718 (258)	2281 (272)	2296 (187)	2937 (446)	4886 (602)	2531 (625)	3882 (649)	0.052 (0.013)	0.076 (0.018)
FRT-like	5	908 (286)	1293 (291)	1481 (236)	1443 (294)	1837 (429)	3535 (627)	1593 (422)	2626 (473)	0.314 (0.088)	0.389 (0.105)
Jump	72	310 (16)	661 (23)	865 (75)	919 (40)	1388 (80)	10912 (1366)	2790 (205)	10601 (1377)	0.551 (0.026)	0.821 (0.031)
Bubbles	13	507 (80)	894 (114)	1168 (167)	1060 (137)	1348 (135)	3719 (648)	980 (143)	3211 (682)	0.194 (0.056)	0.286 (0.091)

Means (and standard error of the mean) of selected acoustic parameters measured for the most common sound types for each species (1094 sounds out of 1197). Characteristics of the air gulp event (surface splash or jump) and individual bubble release sounds are also provided. FRT = fast repetitive tick, VFRT = very fast repetitive tick.

**Fig 3:**
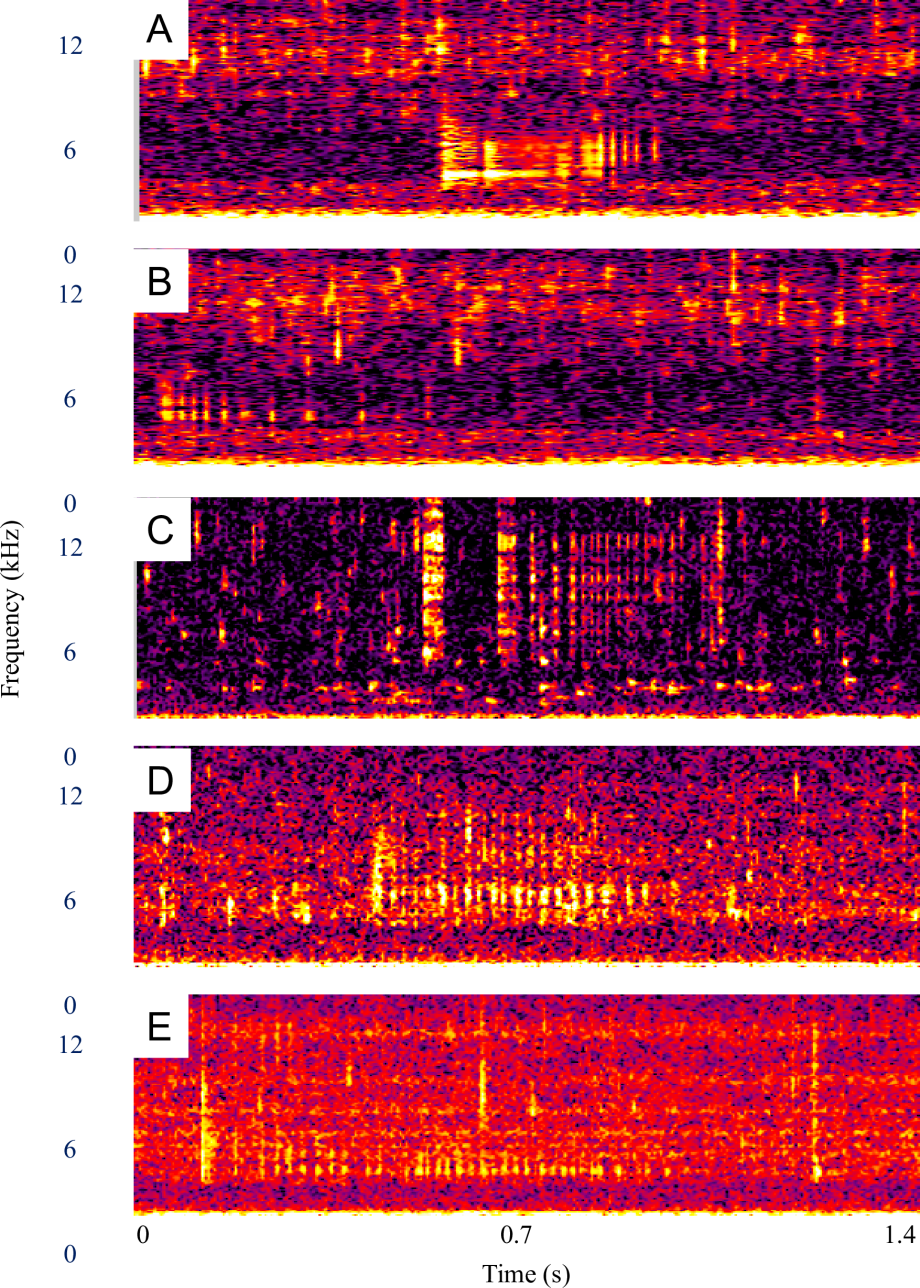
Comparison of “FRT” sounds. (**A) and (B)** Alewife, (**C)** brook trout, (**D)** brown trout, and (**E)** rainbow trout. Spectrograms in each graph are unfiltered and set to the same parameters (1024 Hann windowed FFTs with 50% overlap), time scale (0 to 1.4 s), and frequency scale (0 to 12 kHz).

**Fig 4:**
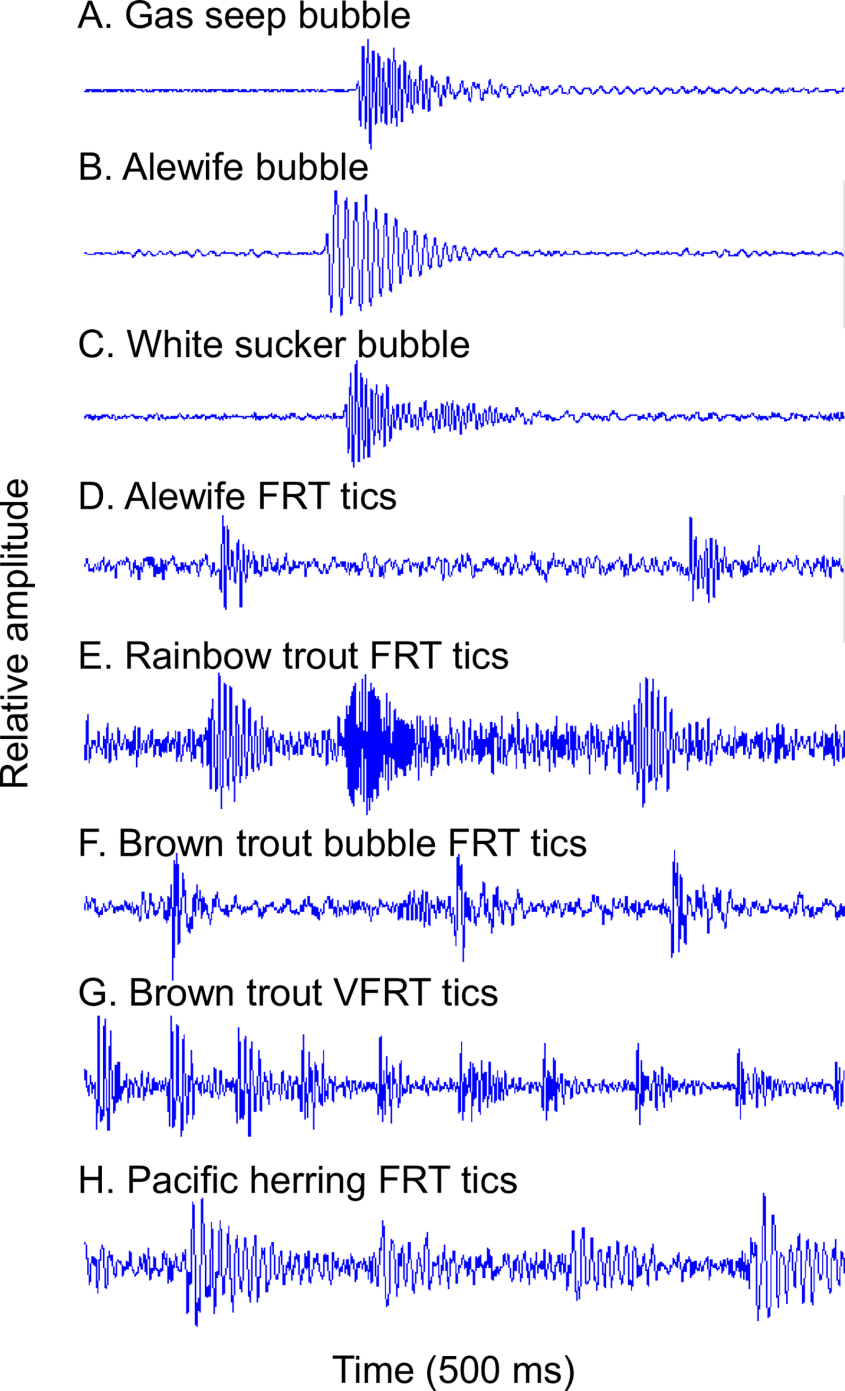
Comparison to bubble sounds. Waveforms of individual air bubble sounds, and individual FRT ticks are compared on the same 500 ms time scale after filtering around 500 Hz to 6000 Hz (amplitudes are relative and not directly comparable). (**A)** Single bubble from bottom sediment gas seep, (**B)** single bubble from alewife gills, (**C)** single bubble from white sucker, (**D)** two ticks from an alewife FRT, (**E)** three ticks from a rainbow trout FRT, (**F)** three ticks from a brown trout bubble FRT, (**G)** multiple ticks from a brown trout VFRT, and (**H)** four ticks from a Pacific herring FRT.

The much less frequently observed snitches (Fig 2C and 2E) had a similar bimodal frequency structure, but were weaker and shorter in duration. FRT-like sounds (examples in Fig 3A and 3B) occurred in only 9% of the sound series (Table 2) and were characterized by a longer duration (mean = 1.3 s) and higher frequency (mean peak frequency = 6,254 Hz, Table 3). Bubble sounds (example Fig 4B) had a higher peak frequency (mean = 1,716 Hz), and shorter duration (mean = 0.009 s), than the coughs and snitches (Fig 2A, Table 3).

### White sucker

Behavior of white sucker was monitored over about 16 h (958 min) across 11 dates at the Stony Brook Herring run, and for an additional 7.5 h (450 min) during spawning events at the Stony Brook Herring run and Webber Pond sites (S1 Appendix). Mill pool held only a few white sucker at any one time. At dusk, sedentary individuals that had been resting on the bottom became more active, and then would rise to the surface and gulp air in a loud splash or jump. They could then be observed to trail bubbles from the mouth and gills as they descended. As with alewife, sound production occurred after the individual had returned to the bottom, or to a level swimming location above the bottom. We did not observe air release during sound production but cannot rule out the possibility due to the difficulty of observing individuals in the limited visibility at dusk and the limited video field of view. White sucker sounds were only heard after a surface event just prior to or after sunset.

During observations of actively spawning white sucker in both the Stony Brook and Webber Pond sites, two to four groups of three to five males and a single large female were observed to repeatedly spawn throughout the late afternoon and well after dark. Thrashing and rattling sounds could clearly be heard as females deposited eggs in the stream or pond bed. At Webber pond, spawning occurred within inches of the shore in still water as shallow as 10–15 cm, while at Stony Brook spawning occurred in fast flowing riffles over gravel. In between spawning bouts, females would move to quieter water and rest on the bottom for a period prior to the next spawn. Males would sometimes accompany the resting female, but some continued to swim actively in the area. Groups of spawning individuals did not exhibit either the air gulping or sound production behaviors even at sunset in contrast to the non-spawning individuals located a short distance away (< 20 m) within the mill pool.

**Fig 5:**
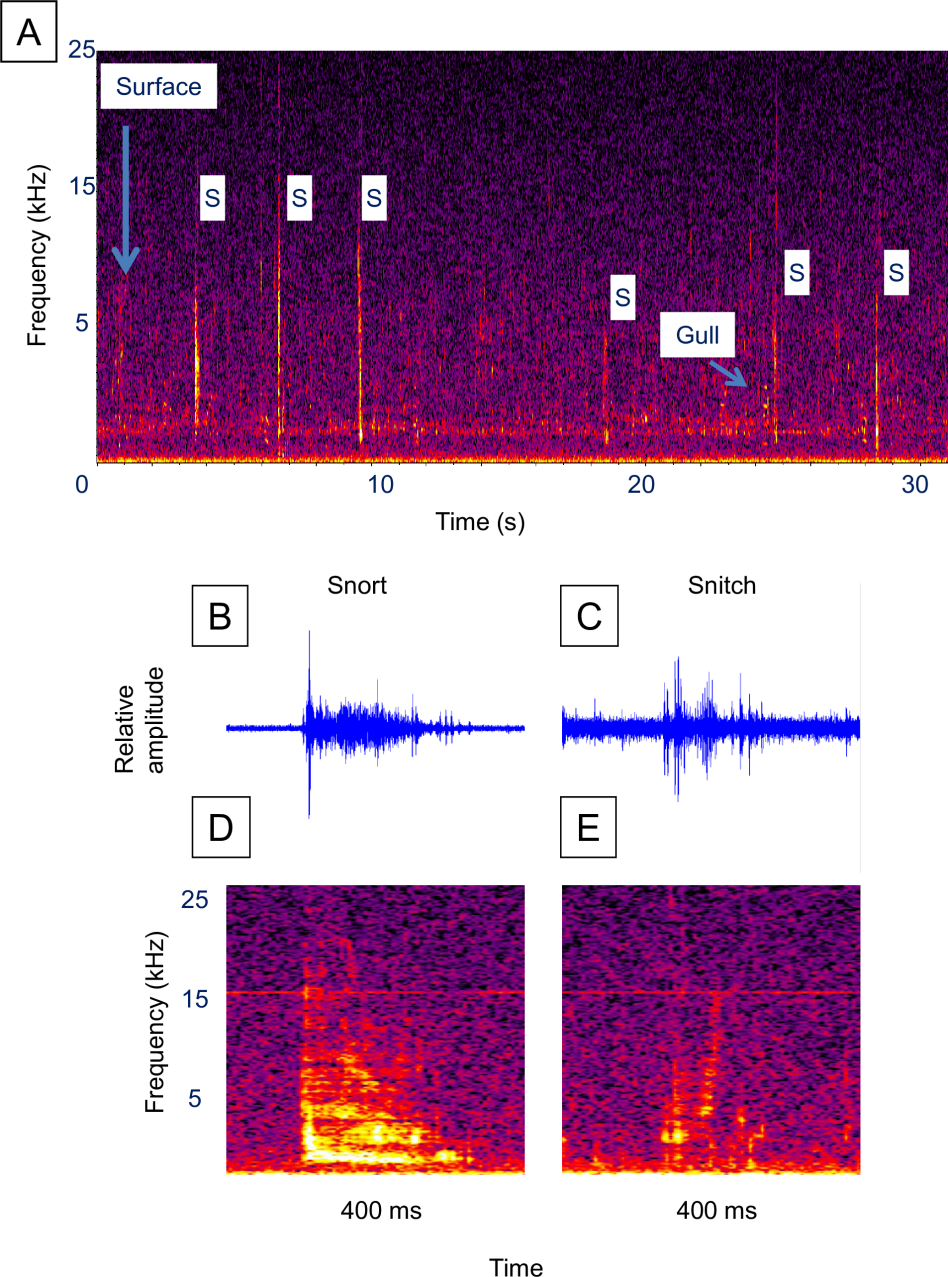
White sucker sounds. Example of a surface event sound series and the two most frequently observed fish sounds. (**A)** Spectrogram of a surface event sound series (see S2 Audio), (**B)** waveform of a snort, (**C)** waveform of a snitch, (**D)** spectrogram of the snort in **B**, and (**E)** spectrogram of the snitch in **C**. Figure labels: “Surface” = air gulping surface event, “S” = snort, “Gull” = herring gull sound heard underwater. Spectrogram parameters: unfiltered, 1,024-point Hann windowed FFTs with 50% overlap. Fish sound waveforms were filtered around 700 Hz to 13000 Hz.

White sucker produced 3 to 11 loud “snort” sounds (mean = 7) with a latency of 2.6 s after the surface event, and a mean fish sound series duration of 16.75 s (Fig 5A–5E, Table 1, S2 Audio). Snorts occurred in all white sucker sound series, while snitches occurred in 33% (Table 2). Surface event sounds were detected in 92% and bubble sounds in 58% of the sound series. Snorts had a mean peak frequency of 1,954 Hz, mean bandwidth of 12,285 Hz, and duration of 0.175 s (Fig 5B and 5D; Table 3), while snitches had a mean peak frequency of 2,291 Hz, bandwidth of 10,348 Hz, and duration of 0.083 s (Fig 5C and 5E; Table 3). Bubbles sounds had a mean peak frequency of 1,548 Hz, bandwidth of 2,794 Hz, and duration of 0.014 s (Table 4, Fig 4C). FRT-like sounds occurred in 8% of the sound series (Table 2). No overt reaction of conspecifics was observed to white sucker sounds, but observations were hampered by low visibility and large separation distances among individuals.

**Table 4:** Comparison of common sounds among species.

	Sound type and species included						
	Bubbles	Chirp	VFRT	Gurgle	Snitch	Snort	Surface event
Variables	A,WS,T	BK,BN,R	BK,BN,R	R,T	A,WS,BK,R	WS,T	A,WS,BK,BN,R,T
**Frequency:**							
Low	*A>WS = T	**BK>R	ns	***R>T	***A = WS<BK = R	***WS>T	***BK = R<A = WS = BN = T
Q1	*A = WS>T	**BK = BN>R	ns	***R>T	***A = WS<BK = R	*WS>T	***A = WS = T<BK = BN = R
Peak	ns	* BK = BN>R	ns	***R>T	***A = WS<BK = R	**WS>T	***A = WS = T<BK = BN = R
Center	ns	* BK = BN>R	ns	***R>T	***A<WS = BK = R	***WS>T	***WS = T<A<BR = BN = R
Q3	***A >WS = T	**BK = BN>R	ns	***R>T	***A<WS = BK = R	***WS>T	***WS = T<A<BR = BN = R
High	ns	*BN>BK = R	ns	***R>T	***A<WS = BK = R	***WS>T	***A = T<WS = BK = BN = R
90% Bandwidth	*A >WS = T	**BN>BK = R	ns	***R>T	***A<WS = BK = R	***WS>T	***A = WS = T<BK = BN = R
Bandwidth	ns	**BN >BK = R	ns	***R>T	***A<WS = BK = R	***WS>T	***A = T<WS = BK = BN = R
**Time:**							
90% duration	*A = WS<T	*BN>BK = R	ns	ns	**A<WS = R<BK	ns	***T>A = WS = BR = BN = R
Duration	***A<WS<T	*BN<BK = R	ns	ns	***A<WS = BK = R	ns	***T>A = WS = BR = BN = R
MANOVA (p)	***	*	**	***	***	***	***

Analysis of variance (ANOVA) comparison of sound parameters among species for selected common sound types. Variables were normalized by appropriate transformations indicated by a maximum likelihood test. In a few cases statistical outliers were omitted. For variables with significant among species main effects a SNK means comparison tested for specific differences among species (for example BK = BN>R indicated that the mean for rainbow trout was significantly less than for brown and brook trout which were not different). Because some species did not exhibit a sound type, or sample sizes were too low, species included in the ANOVA for each type are indicated under the type. MANOVA indicates the results of a multivariate comparison among species based on all variables. A = alewife, WS = white sucker, BK = brook trout, BN = brown trout, R = rainbow trout, T = unknown salmonid. Q1 = mean first quartile frequency, Q3 = mean 3rd quartile frequency.

* = p < 0.05

** = p < 0.01.

*** = p < 0.001.

ns = nonsignificant.

### Brook trout

**Fig 6:**
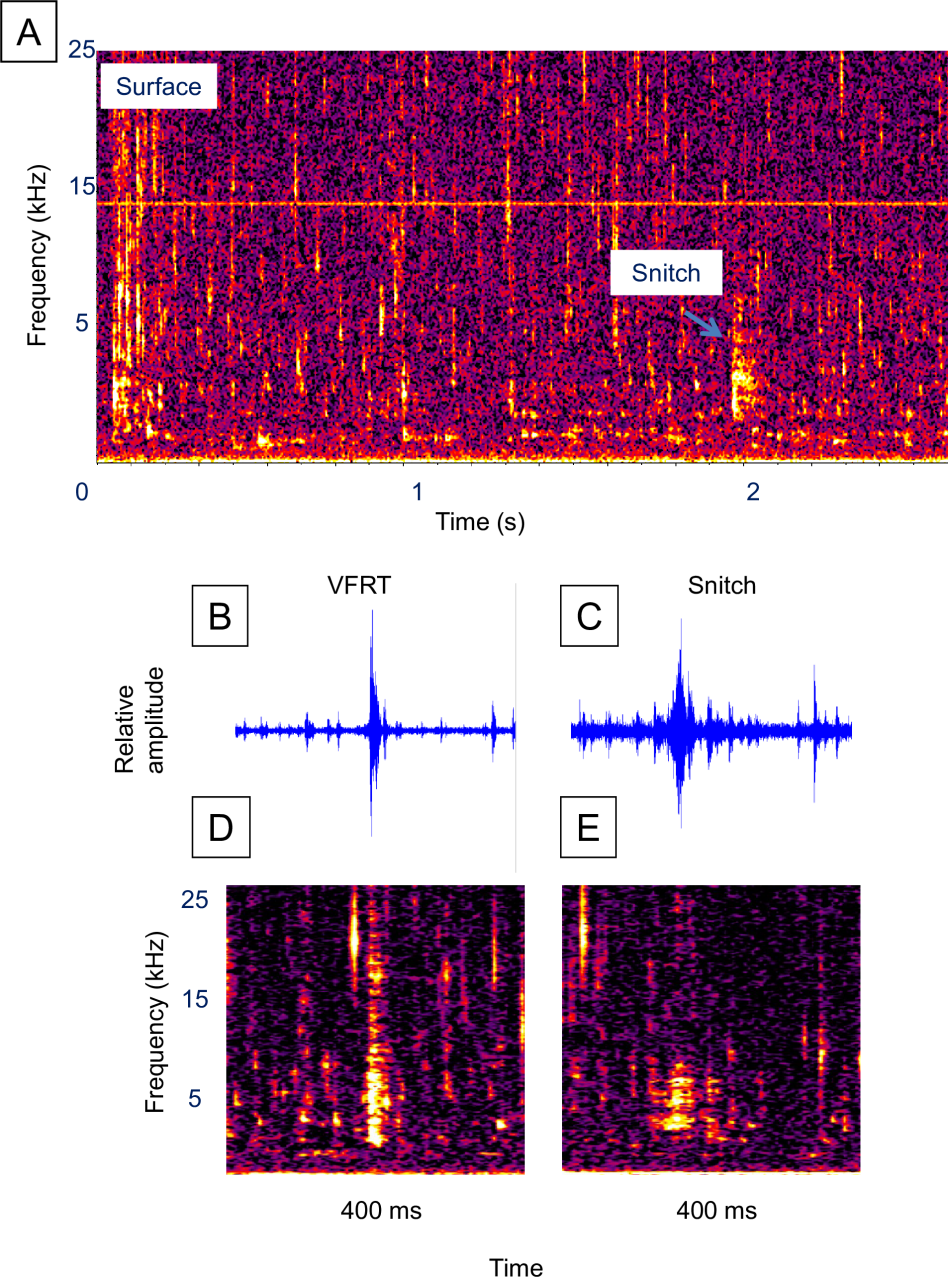
Brook trout sounds. Example of a surface event sound series and the two most frequently observed fish sounds. (**A)** Spectrogram of a surface event sound series (see S3 Audio), (**B)** waveform of a FRT, (**C)** waveform of a snitch, (**D)** spectrogram of the FRT in **B**, and (**E)** spectrogram of the snitch in **C**. Figure labels: “Surface” = air gulping surface event. Spectrogram parameters: unfiltered, 1,024-point Hann windowed FFTs with 50% overlap. Fish sound waveforms were filtered around 2300 Hz to 13000 Hz.

Brook trout were observed for 5.4 h (322 min) over 3 dates in December 2014 (S1 Appendix). Brook trout sounds were infrequent despite the high stocking density in the raceway. Only 6 sound series with one to three fish sounds and a 6 s latency, were positively identified due to the high fish density and limited camera field of view in the raceway (Tables 1 and 2, Fig 6A, S3 Audio). Fish were observed to surface and expel air from gills as they descended followed by sounds after the fish had returned to the bottom, or its original swimming position in the water column. Gas release was not observed in association with sounds but cannot be ruled out due to the difficulty of following individuals after the air gulp event. The most frequently observed sound was a short duration FRT-like sound we term a “Very Fast Repetitive Tick” (VFRT) with a mean peak frequency of 4,993 Hz, bandwidth of 10,407 Hz, and duration of 0.096 s (Tables 2 and 3, Fig 6B and 6D). The next most common sound was named a “snitch” which had similar properties as the VFRT except for a narrower bandwidth of 5,166 Hz and more burst-like waveform lacking the repetitive ticks (Tables 2 and 3, Fig 6C and 6E). Although we observed bubble release immediately after surfacing, brook trout bubble sounds were not acoustically detected, and no bubbles were observed in association with any brook trout sound. No overt reaction of conspecifics to fish sounds was observed.

### Brown trout

**Fig 7:**
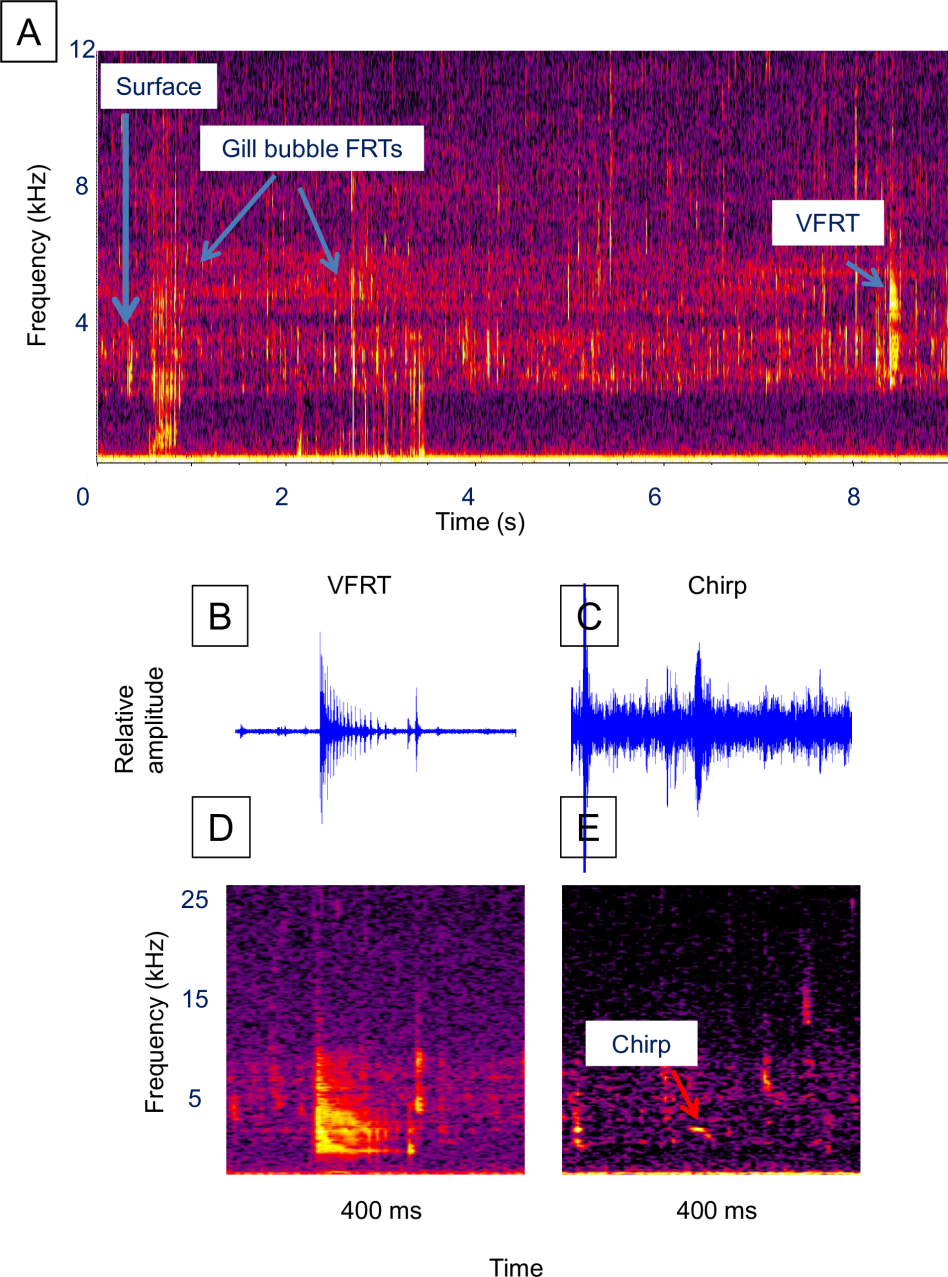
Brown trout sounds. Example of a surface event sound series and the two most frequently observed fish sounds. (**A)** Spectrogram of a surface event sound series (see S4 Audio, S6 and S7 Videos), (**B)** waveform of a FRT, (**C)** waveform of a chirp, (**D)** spectrogram of the FRT in **B**, and (**E)** spectrogram of the chirp in **C**. Figure labels: “Surface” = air gulping surface event, “Gill bubble FRT” = sound of rapid bubble release from gill covers. Spectrogram parameters: unfiltered, 1,024-point Hann windowed FFTs with 50% overlap. Fish sound waveforms were filtered around 2300 Hz to 13000 Hz.

A total of 5.8 h (348 min) of observations of brown trout were made on two dates in December 2014 (S1 Appendix). Additional video observations were recorded on 25 October 2016. Brown trout were stocked at a similar density to the brook trout but produced many more sounds. Air gulping and sound production appeared to increase at dusk. Typically, individuals would rise slowly from a bottom resting position, or midwater swimming position, before accelerating slightly to gulp air usually producing little splash or sounds. Only 4 surface events were acoustically detected out of 26 fish sound series (Table 1). Diffuse streams of air bubbles of varying size could sometimes be observed escaping from the mouth and gills as the fish descended, but such gas release usually did not produce detectable sounds (example S5 Video). However, a unique sound we term a “gill-bubble FRT” was recorded on several occasions simultaneously with bubble release as a brown trout dove from the surface (Fig 7A, S4 Audio, S6 and S7 Videos). Unlike the diffuse bubble streams usually observed during the dive for brown trout, as well as all other species, the FRT-like sound was produced by uniform sized bubbles in a single file that rapidly streamed from either the right or left gill cover, but not both. Shortly after settling, individuals were sometimes observed to simultaneously release one to a few bubbles from each gill, often with a yawn which may have been coincident with VFRT sound production. We refer to this event as a “double gill bubble release”. One individual was observed to surface and produce sounds twice within a 45 s period.

Brown trout sound series averaged 2.2 fish sounds, a 3.3 s latency, and were 1.3 s in duration (Table 1, Fig 7A, S4 Audio, S6 and S7 Videos). A well-defined VFRT was the most frequently occurring sound occurring in 96% of the sound series (Fig 7B and 7D, Table 2), and was characterized by a mean peak frequency of 4,582 Hz and bandwidth of 9,692 Hz (n = 216, Table 3). In several instances, the VFRT appeared to be produced just prior to the double gill bubble release event, but more data are needed to confirm this. A bird-like chirp (Fig 7C and 7E) was the second most frequent sound (27%) in the brown trout series (Table 2) and had a peak frequency of 4,760 Hz (n = 9, Table 3). It should be noted that simultaneous recording with a microphone confirms the bird-like chirp sound is produced underwater and not from an aerial source. Bubble sounds were acoustically detected in only 4% of the brown trout sound series (Table 2), and had a mean peak frequency of 1,031 Hz, bandwidth of 9,430 Hz, and duration of 0.016 s (Table 3). No bubble release was ever observed for any sound other than uncommon gill-bubble FRTs and double gill bubble release events. No overt reaction among conspecifics to brown trout sounds were observed.

### Rainbow trout

**Fig 8:**
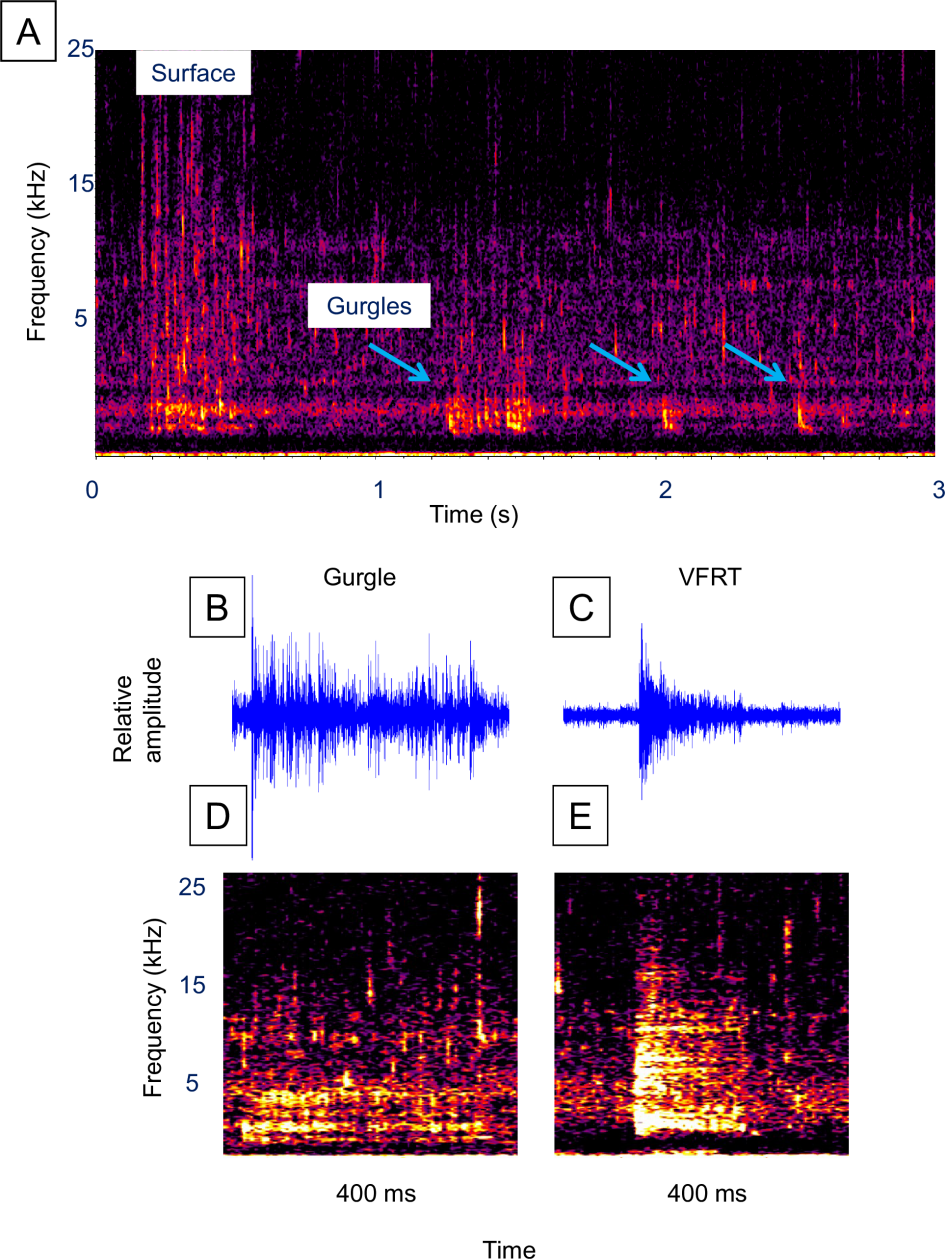
Rainbow trout sounds. Example of a surface event sound series and the two most frequently observed fish sounds. (**A)** Spectrogram of a surface event sound series (see S5 Audio), (**B)** waveform of a gurgle, (**C)** waveform of a FRT, (**D)** spectrogram of the gurgle in **B**, and (**E)** spectrogram of the FRT in **C**. Figure labels: “Surface” = air gulping surface event. Spectrogram parameters: unfiltered, 1,024-point Hann windowed FFTs with 50% overlap. The gurgle waveform was filtered around 1400 Hz and 3900 Hz, while the FRT waveform was filtered around 2000 Hz to 12000 Hz.

Rainbow trout were observed for 3.2 h (193 min) over two dates in December 2014 (S1 Appendix). Unlike brown and brook trout, surface events were acoustically detected for all sound series (N = 42, Tables 1 and 2). Rainbow trout sound series averaged 1.93 fish sounds, a duration of 1.1 s, and a latency of 1.7 s (Table 1, Fig 8A, S5 Audio). The most frequently observed sound was described as a “gurgle” which occurred in 55% of the series (Table 1), and had a peak frequency of 2,409 Hz, bandwidth of 2,468 Hz, and duration of 0.34 s (Table 3, Fig 8A–8D, S5 Audio). A VFRT similar to that observed in brook and brown trout occurred in 17% of the series (Table 2, Fig 8C and 8E), and had a mean peak frequency of 4,199 Hz, mean bandwidth of 10,686 Hz, and mean duration of 0.13 s (Table 3). No bubble release was observed for any sound type. No overt reaction by conspecifics to rainbow trout sounds were observed.

### Unidentified salmonid

Sounds of an unidentified salmonid were recorded in the Presumpscot River over a 3.6 h period on 29 May 2014 (S1 Appendix). Small groups of brook trout were observed among the rocks by an observer from a 2 m high vantage point directly overlooking the site and with a video camera (S1 Appendix). No other species was observed on camera, and several fly fishermen in the area indicated they had only caught brook trout. Occasionally brook trout could be observed from shore as they rose to the surface to gulp air with a small splash and then returned to the bottom. Air bubbles could sometimes be observed flowing from the fish as it descended. Sounds followed after the individual returned to the river bed. Unfortunately, we were not able to capture a surface event on the underwater video camera. At dusk, unidentified salmonids began to periodically jump creating a loud splash followed by gurgle sounds and an unusual moan sound. Individuals observed while it was still light appeared to be salmonids but could not be positively identified. Jumps and associated sounds increased in frequency after dark.

**Fig 9:**
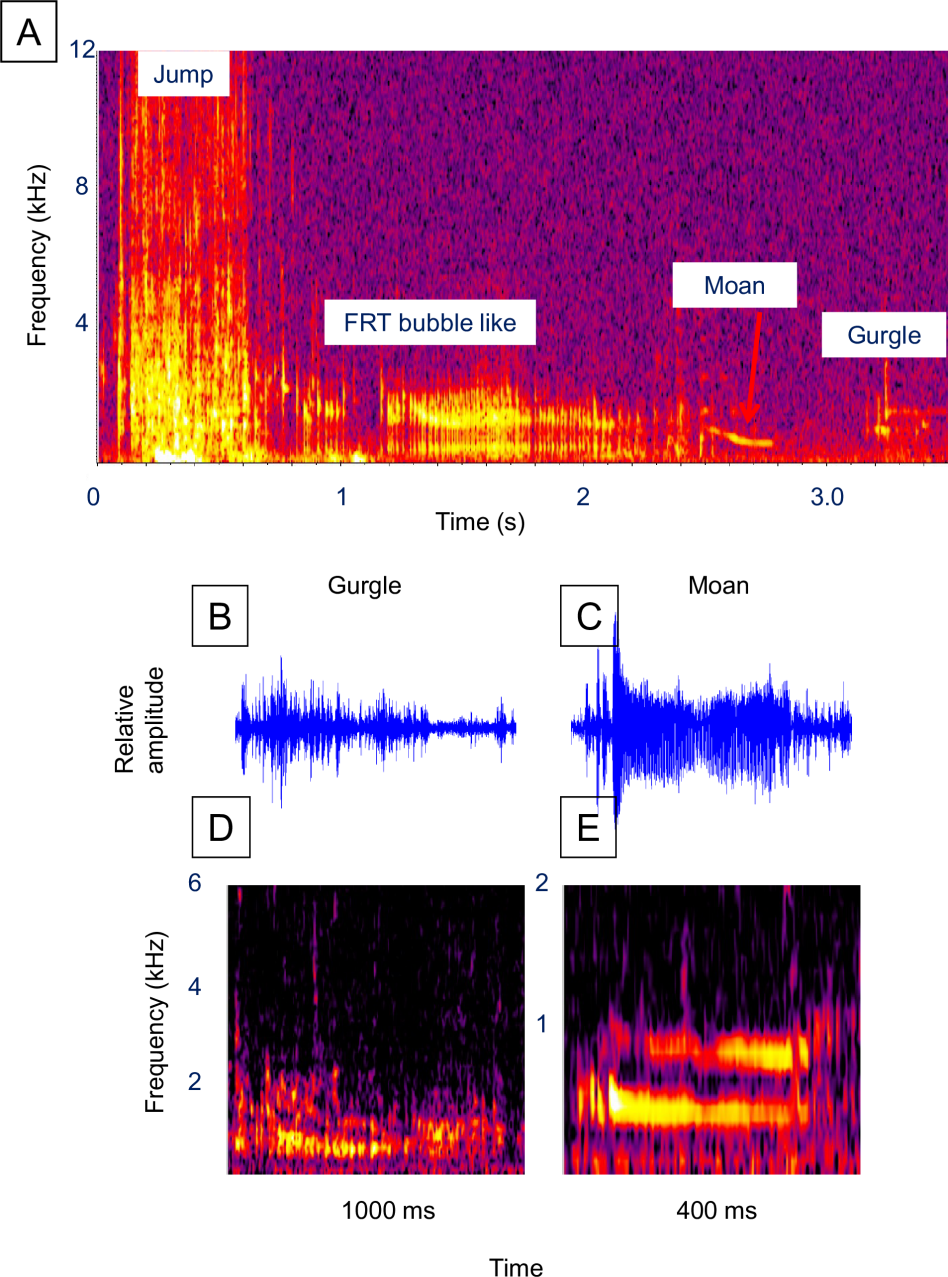
Unknown salmonid sounds. Example of a surface event sound series and the two most frequently observed fish sounds. (**A)** Spectrogram of a surface event sound series (sound S6 Audio), (**B)** waveform of a gurgle, (**C)** waveform of a moan, (**D)** spectrogram of the gurgle in **B**, and (**E)** spectrogram of the moan in **C**. Figure labels: “Jump” = air gulping surface event involving a leap from the water. Spectrogram parameters: unfiltered, 1,024-point Hann windowed FFTs with 50% overlap. The gurgle waveform was filtered around 300 Hz and 3000 Hz, while the moan waveform was filtered around 200 Hz to 2000 Hz.

Surface events were exclusively jumps and were acoustically detected in 85% of 41 detected sound series (Tables 1 and 2). Sound event series were characterized by an average of 2.9 sounds after a 1.6 s latency (Table 1, Fig 9A, S6 Audio). Gurgle (63%) and moan (34%) sounds were the most frequently observed sound types and had mean peak frequencies of 748 Hz and 962 Hz, respectively (Tables 2 and 3, Fig 9). Bubble sounds were detected in 24% of the sound series (Table 2).

### Statistical comparison of sounds among species

There were strong qualitative differences in air movement sounds across species. Some sound types were unique to particular species: coughs to alewife, and moans to the unidentified salmonid. The VFRTs were only produced by brook, brown and rainbow trout, and gurgles only by rainbow trout and the unknown salmonid. When the entire surface event sound series typical of a species is considered, the differences are pronounced. We found significant species differences in the surface event series and fish sound series attributes (Table 1). White sucker had significantly higher event series duration, latency, sound series duration, and fish sound period, and lower fish sound rate, than all other species. The number of fish sounds in a sound series was higher for white sucker and alewife than all other species. Note that among species differences for surface event series duration and latency were not tested due to the low sample size, and fish sound period was omitted because it is statistically redundant with fish sound rate.

**Fig 10:**
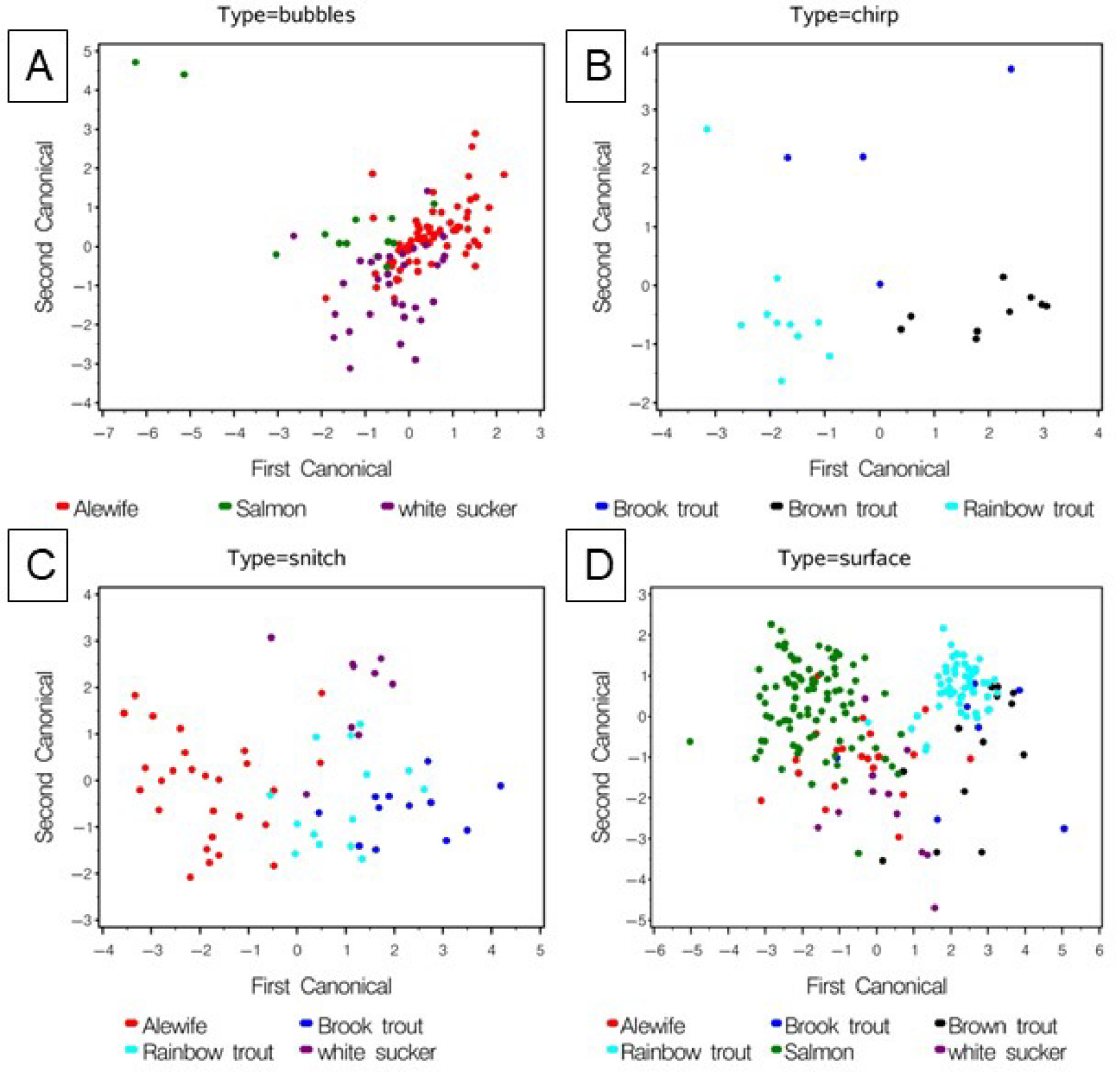
Comparison of selected sounds among species. Canonical discrimination analysis of species groups for selected variables (see Table 5 for canonical statistics): (**A)** differences among species based on bubble sound parameters; (**B)** species differences based on chirp sound parameters; (**C)** species differences based on the snitch sound parameters; (**D)** species differences based on the surface event sound.

A few of the most common sound types, bubbles, chirps, VFRTs, gurgles, snitches, snorts and surface events (= jumps + splash + surface) were tested for differences in univariate and multivariate acoustic parameters among the species which exhibited them (in some cases a species may have been dropped due to a low sample size, Table 4). The MANOVA indicated significant difference among species for all sound types tested (Table 4). Multivariate CDA analysis resulted in significant discrimination among species for bubble, chirp, snitch and surface sound types (Table 5, Fig 10A–10D). Bubble sounds were only weakly discriminant among species (Fig 10A). Differences in the Q1 frequency, 90% bandwidth, 90% duration and duration drove a significant discrimination in chirp sounds among the three trout species (Table 5, Fig 10B). The 5% and 95% frequency percentiles, IQR duration, and duration drove a significant discrimination among four species (Fig 10C). Only surface events could be compared among all species (Table 5, Fig 10D). Brown, brook and rainbow trout tended to separate from alewife, white sucker and the unknown salmonid, with weaker separation among unknown salmonid, alewife and white sucker (Table 5, Fig 10D). The statistical discrimination among species in their surface sounds was driven most strongly by the 95% frequency percentile, 90% bandwidth, and duration (Table 5). The unknown salmonid surface event was significantly longer in duration than all other species (Table 4). No significant univariate differences among VFRT sounds of the three known trout species were observed (Table 4).

**Table 5:** Results of canonical discrimination analysis.

	Bubble sounds		Chirp sounds		Snitch sounds		Surface sounds		All sounds	
Variable	Can1	Can2	Can1	Can2	Can1	Can2	Can1	Can2	Can1	Can2
Low frequency							0.32	0.45		
5% frequency					0.80	-0.43	0.42	0.52	0.74	0.35
Q1 frequency	0.62	0.30					0.76	0.21	0.85	0.33
Center frequency									0.91	0.26
Peak frequency	0.43	0.29							0.86	0.32
Q3 frequency			0.80	ns			0.84	0.18		
95% frequency	0.65	ns					0.91	0.15	0.84	0.20
High frequency					0.71	0.38	0.81	ns	0.67	0.19
IQR bandwidth							0.74	0.25		
90% bandwidth	0.42	0.22	0.63	-0.49			0.87	0.15	0.69	0.11
Bandwidth							0.78	ns	0.57	0.11
IQR duration			ns	ns	0.50	ns			-0.34	0.52
90% duration	-0.66	0.33	-0.47	ns					-0.41	0.67
Duration	-0.72	0.39	-0.50	ns	0.78	ns	-0.53	0.44	-0.43	0.73
Canonical R^2^	0.34	0.28	0.78	0.50	0.75	0.41	0.78	0.40	0.65	0.59
Proportion of variance (%)	57	43	78	22	80	18	72	14	71	22
Canonical P<	0.0001	0.0001	0.0001	0.0149	0.0001	0.0001	0.0001	0.0001	0.0001	0.0001
Species effect P<	0.0001		0.0001		0.0001		0.0001		0.0001	

Results of Canonical discrimination analyses (CDA) among species for selected sound types based on transformed sound measurements. Blank fields indicate the variable was not included in the analysis. Species effect = significance level for a multivariate difference among species. ns = variables that were included in the model but were not significantly correlated with the canonical. Q1 = mean first quartile frequency, Q3 = mean 3rd quartile frequency, IQR = interquartile.

**Fig 11:**
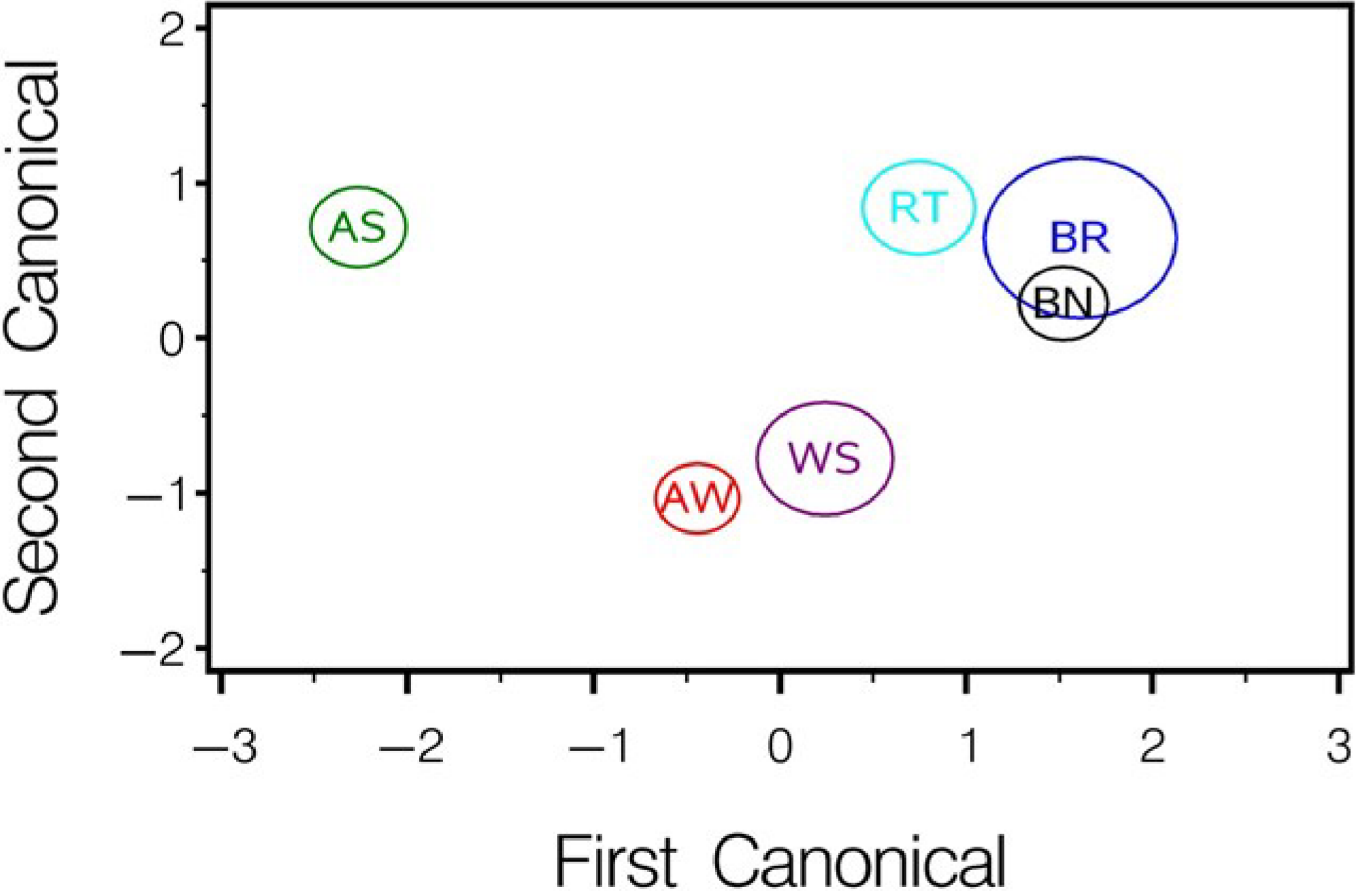
Comparison among species based on all sounds. Canonical discrimination analysis of species groups based on transformed variates for all sound types. The mean canonical and 95% confidence boundary for the mean are shown. Variables that loaded highest on the canonicals, and canonical statistics are provided in Table 5. AW = alewife, BR = brook trout, BN = brown trout, RT = rainbow trout, WS = white sucker, and SA = unknown salmonid.

When data are pooled over all sound types, CDA analysis reveals strong discrimination among species groups (Table 5, Fig 11). The first canonical explains 71% of the variance while the second only 22%. Center, peak and first quartile (Q1) frequency (positive) and duration and 90^th^ percentile bandwidth (negative) drive the discrimination along the first canonical axis. As might be expected, brook and brown trout overlap but discriminate from rainbow trout, all three are strongly different from alewife and white sucker. Perhaps unexpectedly, the unknown salmonid discriminates strongly from the three trout species.

### Bubble comparison

The comparison of the pulse structure of air bubble sounds with individual ticks in the various FRT-like sounds indicates a strong similarity (Fig 4). Both bubbles and FRT ticks show a similar damping oscillation pattern. However, alewife cough and snitch sounds, and trout gurgle and chirp sounds do not appear similar to bubbles in their pulse structure (compare Fig 4 with Figs 2B and 2C and 7C and 8B). White sucker snort and snitch sounds (Fig 5B and 5C) show some similarity to bubbles, but the relationship is uncertain.

## Discussion

This study represents the first descriptions of sound production in the alewife, the white sucker, the brook trout and the brown trout. Limited descriptions of rainbow trout sound production have previously been reported [16–18]. These five species belong to three different orders of fishes: Clupeiformes, Cypriniformes, and Salmoniformes, respectively, suggesting that air movement sound production is widespread within physostomous fishes.

### Alewife

The most frequently recorded alewife sounds in this study were the “cough” and “snitch”. The cough trains produced by alewife after gulping air (Table 4) are not similar to sounds reported for other clupeiform fishes which typically primarily produce FRTs [5, 7–8, 19]. However, alewife do occasionally (9% of the sound series) produce FRT sounds similar to those reported for *Clupea* spp. and *Sardinops sagax*. Although the mechanism by which the cough and snitch sounds are produced is uncertain, it appears that each cough is produced within the pharyngeal or branchial chambers just prior to emission of one or more gas bubbles from one or both gill covers. However, it is clear that release of gas through the gills or mouth alone does not produce sounds similar to either the coughs, snitches or FRTs (compare S1 and S2 Videos exhibiting strong cough sounds, with S3 and S4 Videos where bubbles are released with almost no detectable sound). We suggest that the air bolus in the pharyngeal or branchial chambers amplifies stridulation sounds produced internally within the chambers in a way similar to that hypothesized for the croaking tetra (*Glandulocauda inequalis* = *Mimagoniates inequalis*, Characidae, Characiformes) [20]. Gas release and sound production do not appear to be caused by changes in pressure with depth, as most sounds are produced after the fish has returned to its previous swimming depth and in very shallow water.

Although we did not observe overt reactions of conspecifics to alewife sounds, the sounds were well within the species’ hearing range [21–23]. The observations that sounds are only produced after some air gulp events could be interpreted as possible evidence for voluntary sound production, or it could simply be that the fish sometimes has to expend more effort to expel the gas (i.e., it is literally an involuntary cough to expel gas trapped within the gill chamber).

### White sucker

Our observations of air movement sound and associated behavior for white sucker are similar to those reported for the Eastern creek chubsucker (*Moxostoma oblongus*, Catostomidae; [24]. Chubsucker were reported to produce four or five sounds in a single bout, whereas white sucker were found to produce sounds in bouts of 3 to 11 snorts in this study (Table 1). Abbot [24] described chubsucker sounds in association with air-bubble discharge, however, it is not clear if they were coincident with bubble emission or occurred after bubble release as in the white sucker.

The only other sounds known to be produced by catostomids are those of redd cutting during spawning [25]. River redhorse (*Moxostoma carinatum*, Catostomidae) and robust redhorse (*Moxostoma robustum*, Catostomidae) spawning activities were recorded in Georgia (USA), and the authors proposed that passive acoustic surveys could be used to document spatial and temporal distribution patterns of spawning for these and other endangered or threatened catostomids based on the redd cutting sounds. Their failure to report air movement, or air gulping behaviors, for the two redhorse species is consistent with our observations that white sucker do not exhibit these behaviors during active spawning.

### Salmonids

**Fig 12:**
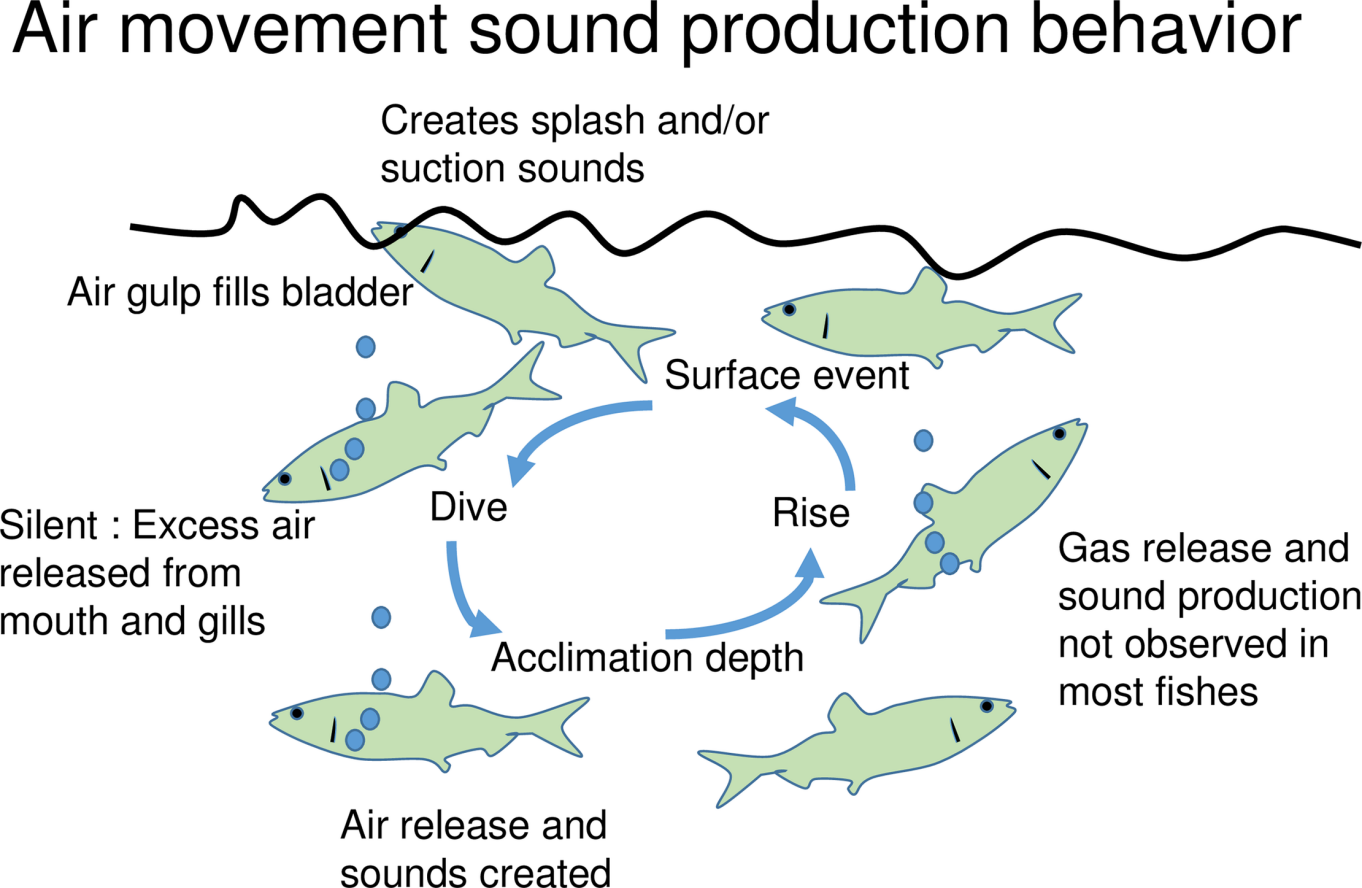
Sound production behavior. Schematic illustration of generalized air movement sound production behavior.

The behavior of the trout and unidentified salmonid observed in this study is similar to that observed for other salmonids [6, 8, 9, 17]. Stober [6] notes that a visible trail of bubbles was emitted from the gill of cutthroat trout, *Oncorhynchus clarkii*, as they dove after rising to the surface and creating a splash. Squawks (600–800 Hz) usually occurred several seconds after the fish had returned to its original position, which is very similar to our observations for brook, brown, rainbow trout, and the unidentified salmonid. Similarly, the behaviors associated with Arctic charr (*Salvelinus alpinus*) sound production [9] are similar to those observed for trout in this study and agree with the general pattern of air movement sound production described herein (Fig 12).

The unidentified salmonid sounds recorded from the Presumpscot River are provisionally attributed to landlocked Atlantic salmon (*Salmo salar*, Salmonidae) which are abundantly stocked at the sampling location. The attribution to Atlantic salmon is supported by similarities with other salmon species and significant differences with the three trout species. The qualitative description of salmon sounds (*Salmo* and *Oncorhynchus*) provided by Neproshin and Kulikova [17] were similar to the unidentified salmonid sounds. They reported that salmon make sounds in the form of splashes when emerging from the surface followed by croaking, rumbling and whistle (similar to our moan) noises from air passage. More recently Kuznetsov [8] presents data from chum salmon (*Oncorhynchus keta*, Salmonidae) and pink salmon (*Oncorhynchus gorbuscha*, Salmonidae). He reports a sound duration of chum salmon of 250–1,750 ms, and peak frequency in two modes at 100–330 Hz and 450–740 Hz. Pink salmon had shorter sound durations of 420–800 ms, and slightly higher peak frequency modes of 200–400 Hz and 420–950 Hz. The dominant frequency of Atlantic salmon is similar to that reported for chum and pink salmon [8], and significantly lower than that of the three captive trout species we recorded (Tables 3 and 4). Further, although brook trout were also abundant at the Presumpscot River site, the behavior and sounds of captive trout were different from those observed for the unidentified salmonid. First, the dramatic moan sounds were unique to fish in the river. Second, the gurgle sounds produced by rainbow trout were of significantly higher frequency than the unidentified salmonid gurgles, and rainbow trout are not stocked in the river. Lastly, there was a clear change in behavior of the unidentified salmonid at dusk with individual fish beginning to jump. Although surface events did appear to increase at dusk for the captive trout, the increase was not as dramatic as in the river, and jumping was not common. However, since sound production of Atlantic salmon has not been previously reported, further research is needed to confirm the river fish sounds recorded in this study were indeed made by the species.

The potential of PAM for behavioral, ecological, and conservation monitoring studies is increasingly being recognized, and applied both to purposeful sounds as well as incidental sounds produced by fishes (see review in [1]). Since air passage sounds appear to be common among Salmonidae, we reiterate previous calls for the use of PAM in field and laboratory studies of salmonids (e.g., [6, 8–9, 26]). Although the observed qualitative and quantitative differences among the sound series of brook trout, brown trout, rainbow trout and Atlantic salmon strongly suggests that each species can be identified in the wild by their sounds, more research is needed to quantify sound characteristics and to confirm species specific characteristics.

We suggest that measurements of the pulse structure of the VFRT sounds (e.g., number of ticks, tick rate, period, frequency, etc.) may be particularly useful for PAM. In addition, several of the observed behavioral and acoustic differences among the salmonid species have potential for use in PAM surveys. All frequency and sound duration parameters exhibited differences among the four salmonid species (Tables 3 and 4, Fig 10C). Multivariate analysis based on data pooled over all sound types suggests that brook and brown trout sound parameters overlap but differ strongly from rainbow trout and Atlantic salmon which were different from each other (Fig 10). Rainbow trout and Atlantic salmon were more likely to make a detectable splash sound than either brook or brown trout, and their most frequent sound was the gurgle. Brown trout were the most prolific sound producers among the captive trout but tended to make quiet air gulps and almost always produced VFRT sounds. Brook trout were the least sound productive but produced similar VFRT and snitch sounds as those of the brown trout.

### Generalized behavior cycle

Based on our observations we can construct a generalized air movement sound production behavior for physostomus fishes that includes a rise to the surface, air gulp event, dive to depth, and resumption of normal behavior at a presumed acclimation depth (Fig 12). Studies of air movement sound production should examine the entire cycle to better understand the behavior. Differences among species in behavior at each step can be informative. For example, differences in the surface event and latency among the three trout species, suggests differences in the physiological process of filling the gas bladder or different adaptations to predation risk at the surface. The presence or absence of gas release during any stage has implications for the sound production mechanism. For example, we did not see evidence of FRT sound production arising from gas release due to pressure changes during either the ascent or descent stages. In fact, most sound production occurred at the end of the dive after the fish had settled to the bottom or assumed a stable swimming depth where it appeared to be neutrally buoyant which we refer to as the “acclimation” depth.

### What is a FRT?

The results of this study, and the qualitative comparisons of previously reported FRT sounds, reveal a diverse array to the form of FRT-like sounds. In general, FRTs consist of an initial broadband burst pulse where ticks are too close to resolve, followed by a train of ticks with a decaying tick interval and tick bandwidth. However, the initial broadband burst is not always present, and the tick train can be either very fast and short in duration (e.g., the VFRT), or long (as much as 10 s or more). It is also likely that some of the burst pulse sounds we have observed are FRTs that lack the tick train.

The relationship between FRT sound production and air passage is uncertain; some researchers have observed FRT sounds coincident with air emission from the anal vent (e.g., [5, 19, 27]) while others report FRT sounds without air emission [8–9, 17–18, 28]. The qualitative comparison of the waveform of various bubble and FRT sounds suggests that they have a similar pulse structure (Fig 4). Furthermore, the gill-bubble FRT suggests a relationship between bubble release and some kinds of FRT-like sounds that do not arise from air passage from the anal vent. It seems most likely, based on these observations and those of most past researchers, that bubble release is incidental to FRT sound production, sometimes occurring and sometimes absent, and that the sounds are often produced by internal movements of gas. However, more data are needed on the precise timing of the sounds and the air bubble release events. In addition, most authors fail to examine the relationship between air gulping and sound production in any detail; the quantification of the full cycle of air gulping behavior (Fig 12) in relation to sound production would provide important insights to further elucidate FRT production mechanisms, as well as other behaviors related to air gulping such as buoyancy compensation. It is also suggested that the temporal patterns of the FRT sounds (e.g., tick interval decay rate, frequency decay rate) may be useful in species identification, as well as to elucidate interspecies differences in the mechanism of FRT production. Therefore, studies that more clearly define what a FRT is, on the basis of its acoustic properties, and a classification of different FRT types, would be of great aid in studies of fish behavior and contribution to the freshwater soundscape.

### Other types of sounds associated with air gulping

Although the FRT sounds have recently captured the attention of both scientists and the public, they are just one of a wide diversity of air movement sounds that were observed in this and previous studies. All six species observed during this study occasionally produced FRT sounds similar to herring, however, other types of air movement sounds were far more prevalent (including the VFRT). In a companion survey of the soundscape of freshwater habitats in the northeastern United States [4], air movement sounds other than FRTs were an order of magnitude more numerous (0.239 sounds/min) and occurred at more recording locations (45%) compared to FRT sounds (0.028 sounds/min and 24% of locations). Both types of air movement sounds were major components of the biological soundscape comparable to the contribution of insects and other fish sounds (i.e., conventionally recognized stridulation and gas bladder sounds).

The observations of air bubble release from the gills after alewife cough sounds, and possibly after trout VFRT sounds, suggests that the sound originates inside the gill chamber, perhaps by vibrating the gill rakers or other internal structures. The weaker alewife snitch sounds, and frequent observation of a lack of sound production with air bubble release, might result from an insufficient pressure of the moving air bolus over the internal structures. Acoustic similarities between the alewife cough and snitch sounds and the trout VFRT sounds support this hypothesis, as do Nelson’s [20] observations of air movement sound production in the croaking tetra. Thus, if confirmed by further study, a similar mechanism may have evolved independently in at least three distinct orders of fishes.

It is possible that the gurgle sound and the gill-bubble FRT sounds may be related but further data are necessary to evaluate their relationship. It should be noted that in the companion study of the freshwater soundscapes of New England [4], gurgle sounds were very common, but were underestimated due to their similarity to highly variable sounds of gas seeps from the sediment. Quantification of acoustic differences between gas seeps and air movement sounds like the gurgle and gill-bubble FRT are necessary before investigators can use passive acoustics to map spatial and temporal distribution patterns of these types of sounds. Moans of the unknown salmonid are among the most unique sounds produced by fishes, and thus, if further research confirms the hypothesis that they are produced by Atlantic salmon, they may be useful in PAM studies of Atlantic salmon behavior.

Although not a type of air movement sound, splash and jump sounds associated with the air gulping behavior were among the most discriminant among species, suggesting that the sounds made by fish splashing and jumping, when associated with air gulping, may be identifiable to species in certain circumstances. In addition, the hypothesized communicative function of sturgeon jumping [29] may extend to other species. It is notable that the sounds produced by the surface gulp are broadband and extend into the lower frequencies and may be audible to some fishes. Jumps may be particularly effective as a mechanism to communicate fitness since the airborne duration is likely correlated with swimming speed and power of the individual. Jumping sounds may be considered under the category of percussive sounds, usually produced by bumping or slapping the bottom sediment or hard structures [7] and studies that actually examine jumping/splashing sounds should be undertaken to examine their potential communication function. Unfortunately, few studies have examined jumping behavior, and only Sulak et al. [29] considers sound function. For example, Soares and Bierman [30] recently examined aerial jumping in the Trinidadian guppy (*Poecilia reticulata*, Poeciliidae) and concluded that it was a deliberate strategy for dispersal and dismissed the possibility of a sound function, although they apparently did not examine jump sounds themselves.

Whether fishes can hear air movement sounds is uncertain due to a lack of data on auditory threshold studies of most species. However, the assumptions of similar hearing abilities among species within families are problematic due to high intra- and inter-specific variation, and thus research to quantify hearing in physostomous fishes is critically needed. For example, although some clupeids can hear high frequency sounds, others apparently do not [31]. In fact, *C*. *pallasii* has an upper auditory threshold of about 5,000 Hz indicating that they may hear only the lower frequency components of their FRT sounds. However, Mann et al. [31] conclude that this is sufficient for FRT sound detection. Although an overt reaction of conspecifics to alewife sounds was not observed, the cough and snitch sounds were well within the species’ hearing range [21–23]. The snort sounds of the white sucker were often dramatic against the background noise and are within the hearing range of cyprinids and catostomids [22].

The low upper frequency auditory thresholds of the few salmonids studied to date [32–33] suggest that a social communication function of FRT sounds is unlikely in the group, but other air movement sounds and jump sounds may be audible to some species. Stober [6] tried different conditioning methods on cutthroat trout and found considerable intraspecific variation with startle responses at frequencies as high as 650 Hz but averaged 443 Hz. Some of the salmonid sounds reported in this study, particularly for the unidentified salmonid, had significant energy at these frequencies (Table 5). In a detailed study of Arctic charr spawning behavior and sound production Bolgan et al. [9] concluded that air movement sounds likely did not play a role in communication.

However, even if air movement sounds are in fact incidental in the majority of physostomous fishes, this study suggests that air movement sounds can be used to identify species and thus are a potentially important tool in conservation, resource management and ecological studies utilizing PAM techniques. One of the most important uses of PAM is in studies of animal behavior in the wild. For example, we observed different air gulping behavior among the species ranging from nearly undetectable surface events to vigorous splashing and jumping. Such different behavior might arise from differences in predation risk, or perhaps energy budgets. Differences in latency times and sound production might provide information on physiological adaptations for gas bladder inflation or deflation. The use of PAM to identify habitat and locations where fish are present is especially promising at night and in locations where water depth or clarity render visual surveys ineffective. Studies to determine the frequency of air gulping behavior in individual fish are needed to determine the potential of PAM of air movements sounds for fish census. For example, if individual fish only gulp air once at sunrise and sunset to inflate and deflate their gas bladders, then one fish sound series would represent one fish.

## Supporting information

S1 Appendix Detailed methodology. Detailed description of sampling locations and methods.
(DOCX)

S1 Audio Alewife sounds. Recording of alewife sound series shown in Fig 2A.
(WAV)

S1 Video Alewife sound production behavior. Movie of alewife behavior while producing the sound series shown in Fig 2A.
(MP4)

S2 Video Alewife behavior in slow motion. Movie of alewife behavior while producing the sound series shown in Fig 2A. The video is slowed to half speed to clarify behavior and the relationship between sounds and bubble release.
(AVI)

S3 Video Alewife bubble release. Movie of alewife behavior and bubble release in which only weak bubble sounds were acoustically detected (compare with **S1** Video).
(WMV)

S4 Video Alewife bubble release in slow motion. Movie of alewife behavior and bubble release in which only weak bubble sounds were acoustically detected. Video slowed to half speed to clarify behavior and sounds at the time of bubble release (compare with **S3** Video).
(AVI)

S2 Audio White sucker sounds. Recording of white sucker sound series shown in Fig 5A.
(WAV)

S3 Audio Brook trout sounds. Recording of brook trout sound series shown in Fig 6A.
(WAV)

S5 Video Brook trout sound production behavior. Movie of brown trout behavior during bubble release when fish sounds were not detected.
(WMV)

S4 Audio Brown trout sounds. Recording of brown trout sound series shown in Fig 7A.
(WAV)

S6 Video Brown trout sound production behavior. Movie of brown trout behavior during production of the sound series shown in Fig 7A.
(MP4)

S7 Video Brown trout behavior in slow motion. Movie of brown trout behavior during production of the sound series shown in Fig 7A. Video slowed to half speed to clarify the relationship between bubble release and sound production.
(AVI)

S5 Audio Rainbow trout sounds. Recording of rainbow trout sound series shown in Fig 8A.
(WAV)

S6 Audio Attributed atlantic salmon sounds. Recording of unknown salmonid sound series shown in Fig 9A. Sounds from the unknown salmonid are provisionally attributed to Atlantic salmon.
(WAV)

S1 Data Raw measurement data. Data file containing acoustic measurements for each sound and sound series. Data compiled from Raven Pro 1.5 acoustic software [11] selection tables and edited for clarity.
(XLS)
