# Supplementary material for: Air movement sound production by alewife, white sucker, and four salmonid fishes suggests the phenomenon is widespread among freshwater fishes
Source: PLoS One. 2018 Sep 20;13(9):e0204247. doi: 10.1371/journal.pone.0204247 (PMC6147564; doi:10.1371/journal.pone.0204247)
Supplement: S1 Appendix — Detailed description of sampling locations and methods. (DOCX) [file pone.0204247.s001.docx]

Supplementary S1 Appendix**. Detailed methodology.** Detailed description of sampling locations and methods. For “Air movement sound production by alewife, white sucker, and four salmonid fishes suggests the phenomenon is widespread among freshwater fishes.” Rodney A. Rountree, Francis Juanes and Marta Bolgan. Corresponding author: [rrountree@fishecology.org](mailto:rrountree@fishecology.org) (RR)

## Sampling locations

In Vasselborro (N44° 24.147’, W69° 40.318’) recordings were obtained in three sites; 1) in the Seven Mile Brook (Table A.1, Fig A.1A) where suspected alewife sounds had previously been recorded; 2) in the new Webber Pond Herring Run (Fig A.1B) and, 3) in the adjacent shoreline of Webber Pond (Fig A.1C). Sampling in the Seven Mile Brook was conducted in a wide (<10 m) shallow (< 0.5 m) pool area about 20 m below the Webber Pond dam and the herring run, while the sampling in the new, adjacent Weber Pond Herring Run was conducted in the upper rectangular chamber (ca. 1.5 m x 4 m).

At Marston Mills (N41° 39.082’, W70° 24.891’) recordings were obtained in the upper chamber of the Mill Creek Herring Run (Fig A.1D, ca. 1.5 m x 7 m and < 0.5 m deep).

At Brewster (N41° 44.682’, W70° 06.745’) recordings were collected in a small (ca. 10 m diameter) mill pool which was about 1.5 m deep in the center and sometimes turbid (Fig A.1E) and in the adjacent downstream section of the Stony Brook Herring Run (Fig 1F, < 0.5 m deep).

At Barnstable (N41° 42.137’, W70° 22.833’) recordings of adult trout reared at the Blue Stream Aquaculture trout hatchery under semi-natural conditions were conducted in raceways (1.8 m wide, < 0.5 m deep) and pools (<10 m diameter, <1.5 m deep) constructed in a natural cold-water spring (Fig A.1G). Up to 2000 adult fish were contained in each raceway, while the pools held up to 8000 fish.

Finally, at North Windham (N43° 49.825’, W70° 26.960’) recordings were conducted in the Presumpscot River below Sebago Lake (Fig A.1H) where unknown salmonid sounds had previously been recorded. The Presumpscot River site is a shallow (wadable) area with 1 m to 1.5 m deep holes and crystal-clear water.

The species-specific identity of the sound emitter was validated through direct observation or because observations were carried out where only one species was present. Recording within single species raceways at the Blue Stream Aquaculture insured that sounds could confidently be attributed to each of the three trout species (i.e. brook trout, brown trout or rainbow trout). On the other hand, the mill pool at the Stony Brook herring run contained alewife together with small numbers of various warm-water fishes, including sunfishes (*Lepomis* spp., Centrarchidae), largemouth bass (*Micropterus* *salmoides*, Centrarchidae), yellow perch (*Perca* *favescens*, Percidae), brown catfish (*Ameiurus* *nebulosus*, Ictaluridae) and white sucker. Here, the specific identity of the sound emitter was validated by direct observation of white sucker behavior (i.e., jumping followed by sound production and air bubble release). Alewife were similarly validated by direct visual observation and were the only species present during recording in the Mill Creek and Webber Pond herring runs. The Presumpscot River is heavily stocked with brook and brown trout as well as landlocked Atlantic salmon (*Salmo* *salar*, Salmonidae) in the spring, and the recording location is a popular fly fishing site, but numerous other species may have been present. Sounds were attributed to unknown salmonids only when they followed jumps or surface gulps.

## Acoustic and video data collection

Preliminary data at the Webber Pond (30 April 2012, Table A.1) and at the Stony Brook Herring Run (27April 2012) were recorded with an uncalibrated HTI-96-MIN hydrophone (High Tech Industries, Gulfport, MS; sensitivity = -165 dB re: 1 V/μPa, frequency response: 2 Hz to 30 kHz). Acoustic data were captured at 44.1 kHz and 16 bit with a MOTU Ultralite, bus-powered audio interface connected to a laptop computer using SpectraLab 4.32.18d Professional Sound Analysis software (Sound Technology, Inc., Campbell, CA). All other acoustic data were recorded at 48 kHz (24 bit) prior to 3 May 2014, and at 96 kHz (16 bit) thereafter, by using an uncalibrated SQ26-H1 recorder system with a SQ26-08 Hydrophone (Sensitivity = -169.00 re. 1V/µPa rms, Cetacean Research Technology, Seattle, WA). Voice notes and ambient aerial sounds were recorded to a second channel from a high definition omnidirectional measurement microphone (frequency response 5 Hz to 30 kHz +1/-3dB, model M30BX, Earthworks, Inc., Milford, NH). Underwater video was observed with one or more underwater cameras mounted in a fixed location on the bottom or suspended within the water column. Cameras used included: an Atlantis AUW-5600 underwater video system (Englewood, NJ), an AquaVu AV750CZ fishcam or Av360 Quad camera (Crosslake, Minnesota). Video was captured to either a Sony portable DVD recorder (model VRD‑MC6) or to a laptop personal computer using a USB analog video digitizer (USB-Live 2, Hauppauge Digital, Inc, Hauppauge, New York). The video included acoustic input from the hydrophone and was recorded simultaneously with the sound recordings.

## Acoustic and video analysis

Acoustic and video data were monitored in real-time in the field. Written and oral notes on behavior and sound production were recorded from shore and on a monitor showing the underwater video. It should be noted that sound production behavior was difficult to capture on video, even in the small herring run chambers with clear water, due to the camera’s limited field of view and fixed location. However, an observer looking down at the fish often had a good vantage point to witness fish behavior. Post-processing of acoustic signals was conducted by listening to all recordings in their entirety while simultaneously viewing the sound’s spectrogram (1,024 FFT, Hanning window, 50% overlap) and waveform with Raven Pro 1.5 acoustic software [11]. Sounds were played with the spectrogram zoomed to 0 to 8,000 Hz and by using a 15 s time display but were expanded or zoomed further when necessary. During post-processing, all video recordings were reviewed in their entirety, with special attention to periods when sound production was indicated in field notes or during post-processing of the acoustic data. Slow-speed and frame-by-frame analysis of the video helped elucidate the relationship between sounds and behavior. Acoustic measurements of all sound types were made in Raven of selected parameters following Charif et al. [12]. Frequency parameters included: low frequency (Hz, the lowest frequency exhibited by the sound), peak frequency (Hz; frequency with the highest energy), high frequency (Hz; the highest frequency exhibited by the sound), and bandwidth (Hz; the difference between the high and low frequencies). Frequency and bandwidth percentiles representing the frequency at which the sound is divided by the amount of energy in the percentile were also measured: 5%, Q1 (first quartile), center (frequency at which half the energy is above or below), Q3 (third quartile), 90%, and 95%. The duration (time between the beginning and end of the sound) and 90% duration (the time period containing 90% of the sound energy) were also measured.

## Sampling effort

A total of 56.5 h (3,388 min) of observations were made (Table A.2), in which we measured the acoustic parameters for 1,466 individual sounds. Of these, 1,195 sounds were attributed to the study species; the rest were of unidentified biological sounds, bird sounds, and noises. A total of 160 fish sound series were identified, in which 117 surface events (thus surface event series) were acoustically detected. A total of 404 sounds (including surface event and bubbles) were measured from the 160 fish sound series and used to determine fish sound series attributes and the most common sound types and an additional 791 individual sounds were measured that could not be attributed to a fish sound series but could be used in determining the acoustic parameter statistics for each sound type (i.e., sample size of 404 + 791 = 1,195 sounds total fish sounds measured).


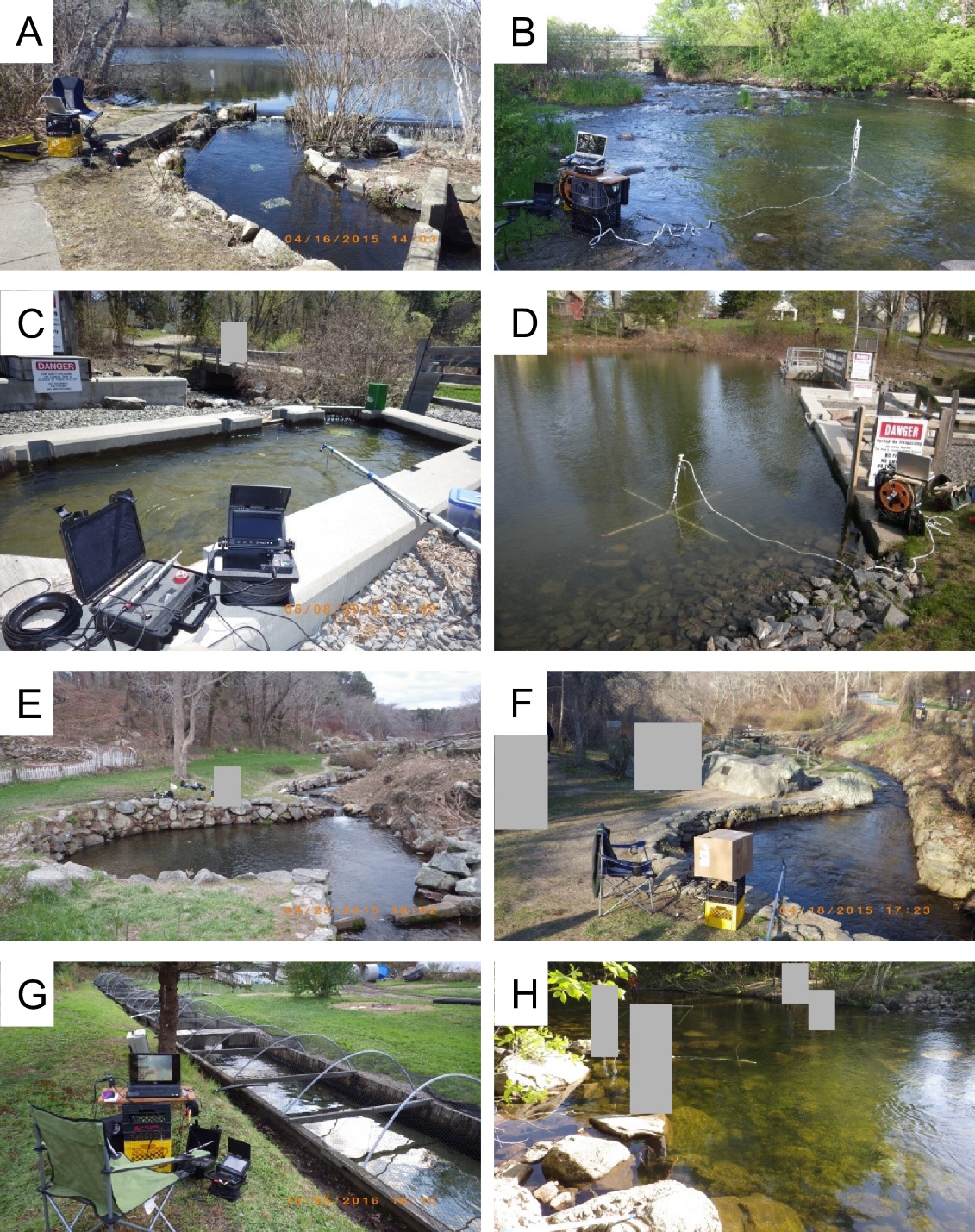


**Fig. A.1. Sampling locations**. A Mill Creek Herring Run in Marston Mills, Massachusetts (alewife); B Seven Mile Brook just below Webber Pond Dam (alewife) in Vassalboro, Maine; C the nearby Webber Pond Herring Run (alewife), D and the adjacent Webber Pond shoreline (white sucker); in Brewster, Massachusetts; E the mill pool in the Stony Brook Herring Run located in Brewster, Massachusetts (alewife and white sucker), F Stony Brook stream just above the mill pool in the Stony Brook Herring Run; G Brook and Brown trout raceways, and rainbow trout pools (not shown) at the Blue Stream Aquaculture trout hatchery in Barnstable, Massachusetts; H Presumpscot River below Sebago Lake in North Windham, Maine. These photographs provide perspective on the spatial layout of the sampling locations, the equipment setup used, and the potential for species identification. Potentially identifiable bystanders in some panels have been obscured.

**Table A.1. Sampling effort for audio recordings and visual observations of fish sound production.**

| **Location** | **Date** | **Start time** | **End time** | **Duration (min)** |
| --- | --- | --- | --- | --- |
| **Alewife** | | | | |
| Mill Creek Herring Run | 22-Apr-14 | 16:22 | 16:40 | 18 |
|  | 24-Apr-14 | 14:25 | 16:23 | 118 |
|  | 2-May-14 | 19:37 | 20:37 | 59 |
|  | 6-May-14 | 13:27 | 17:45 | 258 |
|  | 6-May-14 | 19:48 | 20:56 | 68 |
|  | 12-May-14 | 17:32 | 18:53 | 81 |
|  | 16-Apr-15 | 14:09 | 15:31 | 82 |
|  | 23-Apr-15 | 18:31 | 19:31 | 60 |
| Seven Mile Brook | 28-May-14 | 17:00 | 18:09 | 69 |
|  | 29-May-14 | 11:27 | 11:56 | 29 |
|  | 8-May-15 | 13:59 | 14:26 | 27 |
| Webber Pond Herring Run | 8-May-15 | 12:22 | 12:53 | 30 |
| **subtotal** |  |  |  | **899** |
| **Alewife and White Sucker** | | | | |
| Stony Brook Herring Run | 27-Apr-12 | 19:33 | 20:52 | 79 |
|  | 25-Apr-14 | 19:09 | 19:57 | 46 |
|  | 29-Apr-14 | 15:45 | 18:04 | 112 |
|  | 3-May-14 | 17:43 | 19:00 | 78 |
|  | 7-May-14 | 16:46 | 18:21 | 95 |
|  | 13-May-14 | 17:31 | 18:22 | 51 |
|  | 17-May-14 | 18:30 | 20:47 | 137 |
|  | 20-May-14 | 19:06 | 21:08 | 122 |
|  | 21-Apr-15 | 18:08 | 19:56 | 108 |
|  | 25-Apr-15 | 18:56 | 20:19 | 83 |
|  | 30-Apr-15 | 19:20 | 20:07 | 47 |
| **subtotal** |  |  |  | **958** |
| **Brook trout** | | | | |
| Bluestreams Hatchery | 23-Nov-14 | 16:23 | 16:50 | 26 |
|  | 1-Dec-14 | 16:27 | 17:57 | 90 |
|  | 5-Dec-14 | 15:27 | 18:53 | 206 |
| **subtotal** |  |  |  | **322** |
| **Brown trout** | | | | |
| Bluestreams Hatchery | 1-Dec-14 | 16:27 | 19:30 | 180 |
|  | 5-Dec-14 | 15:52 | 18:43 | 168 |
| **subtotal** |  |  |  | **348** |
| **Rainbow trout** | | | | |
| Bluestreams Hatchery | 1-Dec-14 | 18:00 | 19:30 | 90 |
|  | 5-Dec-14 | 16:02 | 18:30 | 103 |
| **subtotal** |  |  |  | **193** |
| **Unknown salmonid** | | | | |
| Presumpscott river | 29-May-14 | 17:19 | 20:57 | 218 |
| **subtotal** |  |  |  | **218** |
| **White sucker spawning** | | | | |
| Stony Brook Herring Run | 18-Apr-15 | 15:10 | 19:50 | 160 |
| Webber Pond | 30-Apr-12 | 16:16 | 20:31 | 255 |
|  | 8-May-15 | 12:57 | 13:32 | 35 |
| **subtotal** |  |  |  | **450** |
| **Total** |  |  |  | **3388** |

**Table A.2. Number of sounds measured by category.**

| **Species** | **Number of surface event series** | **Number of fish sound series** | **Total sounds** | **Fish sounds** | **Other sounds** | **Fish sounds in a series** | **Fish sounds not from series** | **Sounds used in Table 3** | **Rare sounds excluded from Table 3** |
| --- | --- | --- | --- | --- | --- | --- | --- | --- | --- |
| alewife | 21 | 33 | 347 | 304 | 43 | 90 | 214 | 289 | 15 |
| white sucker | 11 | 12 | 153 | 140 | 13 | 40 | 100 | 126 | 14 |
| brook trout | 4 | 6 | 71 | 56 | 15 | 14 | 42 | 48 | 8 |
| brown trout | 4 | 26 | 306 | 263 | 43 | 47 | 216 | 253 | 10 |
| rainbow trout | 42 | 42 | 226 | 176 | 50 | 96 | 80 | 154 | 22 |
| unknown salmonid | 35 | 41 | 303 | 256 | 47 | 117 | 139 | 224 | 32 |
| total all species | 117 | 160 | 1466 | 1195 | 271 | 404 | 791 | 1094 | 101 |

Fish sounds include surfacing, bubble and other sounds associated with a sound series. Other sounds include incidental sounds such as noises, birds, and sounds not attributed to the target species. Fish sounds not from a fish sound series are those attributed to a species but could not be attributed to a discrete sound series due to temporal overlap, lack of observation, or other uncertainty. Additional fish sounds are rare sounds for which acoustic statistics are not reported in Table 3.
